# Enhancing digestibility and ethanol yield of *Populus* wood *via* expression of an engineered monolignol 4-*O*-methyltransferase

**DOI:** 10.1038/ncomms11989

**Published:** 2016-06-28

**Authors:** Yuanheng Cai, Kewei Zhang, Hoon Kim, Guichuan Hou, Xuebin Zhang, Huijun Yang, Huan Feng, Lisa Miller, John Ralph, Chang-Jun Liu

**Affiliations:** 1 Biology Department, Brookhaven National Laboratory, Upton, New York 11973, USA; 2 Department of Biochemistry, and the DOE Great Lakes Bioenergy Research Center, The Wisconsin Energy Institute, University of Wisconsin, Madison, Wisconsin 53726, USA; 3 Dewel Microscopy Facility, Appalachian State University, Boone, North Carolina 28608-2027, USA; 4 Department of Earth and Environmental Studies, Montclair State University, Montclair, New Jersey 07043, USA; 5 National Synchrotron Light Source, Brookhaven National Laboratory, Upton, New York 11973, USA

## Abstract

Producing cellulosic biofuels and bio-based chemicals from woody biomass is impeded by the presence of lignin polymer in the plant cell wall. Manipulating the monolignol biosynthetic pathway offers a promising approach to improved processability, but often impairs plant growth and development. Here, we show that expressing an engineered 4-*O*-methyltransferase that chemically modifies the phenolic moiety of lignin monomeric precursors, thus preventing their incorporation into the lignin polymer, substantially alters hybrid aspens' lignin content and structure. Woody biomass derived from the transgenic aspens shows a 62% increase in the release of simple sugars and up to a 49% increase in the yield of ethanol when the woody biomass is subjected to enzymatic digestion and yeast-mediated fermentation. Moreover, the cell wall structural changes do not affect growth and biomass production of the trees. Our study provides a useful strategy for tailoring woody biomass for bio-based applications.

## Abstract (web summary)

The efficiency of cellulosic biofuel production from woody biomass is limited by the presence of lignin that impedes efficient processing. Here the authors show that transgenic modification of aspen to depress lignin polymerization can increase ethanol yield without affecting tree growth.

## Graphical abstract

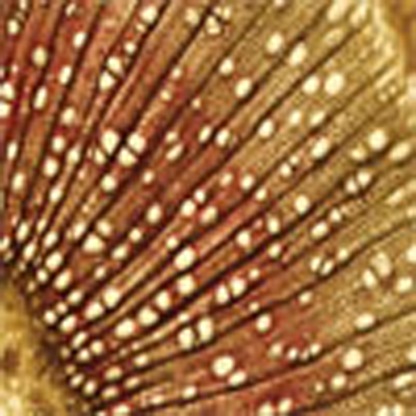

Wood is one of the world's most abundant natural materials. It is widely used in pulp and paper manufacturing and recently has been considered a promising source of simple sugars for industrial bio-based products and advanced biofuels^1^. Wood is the secondary xylem of vascular plants, consisting of a compound middle lamella and secondary wall layers composed of a cellulose/hemicellulose network, and impregnated with lignin. In the wood of *Populus* spp., these polymers occur in the approximate proportions of 45% cellulose, 25% hemicelluloses and 20% lignin^2^. Lignin in the wood forms a hydrophobic barrier hindering the access of digestive enzymes to polysaccharides and decreasing their activity; therefore, it is a significant limiting factor in converting woody biomass to fermentable sugars in processes towards biofuels^1^. Lignins are complex and heterogeneous polymers of 4-hydroxy-phenylpropanoid units^3^. They are derived from the oxidative radical coupling of three typical building blocks (that is, the monolignols), *p*-coumaryl, coniferyl and sinapyl alcohols (Fig. 1a). After their incorporation into the growing lignin polymer, these hydroxycinnamyl alcohols correspondingly produce *p*-hydroxyphenyl (H), guaiacyl (G) and syringyl (S) subunits. The amount and composition of lignin varies among taxa, cell types and the individual layers of the cell walls. In general, the S/G ratio dictates the degree of lignin condensation and reactivity by supporting different types of subunit linkages. A higher G-unit content creates more condensed lignin that is composed of a greater portion of biphenyl, phenylcoumaran and other carbon–carbon linked units as their 5-position on the aromatic ring is available for radical coupling reactions. S-units, in contrast, are more commonly linked through ether bonds at the available 4-hydroxy position of the growing lignin polymer yielding more linear and chemically relatively labile polymers^3^. For several decades, considerable effort has been expended in tailoring monolignol biosynthesis to control lignin content and/or composition. Several studies have successfully demonstrated that reducing lignin content or modifying its structure in plants could translate into improved saccharification efficiency, and thus, lead to reduced biomass conversion costs^4^,^5^,^6^,^7^. A few studies also reported that manipulating monolignol biosynthetic genes such as caffeic acid *O*-methyltransferase (*COMT*), and 4*-*coumarate-CoA ligase (*4CL*) in, for example, swtichgrass (*Panicum virgatum*) or ryegrass (*Lolium pernne*), appeared to have no negative effect on crop's agronomic performance and/or biomass yield^8^,^9^,^10^. However, on the other hand, many investigations of different plant species show that the growth and development of the engineered plants is often compromised by the simple disruption of the monolignol biosynthetic pathway, and/or by a dramatic reduction of the content of lignin in the cell wall^11^. This is particularly evident in the manipulation of hydroxycinnamoyl-CoA: shikimate hydroxycinnamoyl transferase (*HCT*) gene in alfalfa^12^, cinnamate 4-hydroxylase (*C4H*), coumaroyl 3′-hydroxylase (*C3′H*), and cinnamoyl-coa reductase (*CCR*) genes in *Arabidopsis*, alfalfa, and hybrid poplars^6^,^13^,^14^,^15^. Even though down-regulation of *4CL* did not affect biomass production of swtichgrass, the silencing of this gene in pine^16^ and poplar caused obviously stunted plant growth^17^,^18^. Similarly, *COMT*-deficient *brown midrib* mutants of maize (*bm3*) and sorghum (*bmr12*), and *COMT*-downregulated sugarcane exhibited a biomass yield reduction^19^,^20^,^21^. Therefore, it is desirable to further explore a more effective strategy to modulate lignin synthesis and structure, thus improving the efficiency of the bioconversion of wood, while sustaining the plant growth and development.

Lignin polymerization commences with one-electron oxidation of the phenols of monolignols by oxidative enzymes, laccases and/or peroxidases, to yield activated radical intermediates. The coupling of monolignol radicals with each other (for the initiation of lignin polymer) and, more importantly, with the growing polymer forms lignins^3^,^22^ (Fig. 1b). In this process, a free phenol (the unsubstituted 4-hydroxy group of a monolignol, and/or the free-phenolic end of the growing polymer), is essential for generating phenolic radicals to allow radical coupling that forms the different types of lignin inter-unit linkages^3^,^23^. Previously, we demonstrated that chemical modification, that is, the methylation of the 4-hydroxy group of a monolignol, prevents the participation of the monolignol derivative in the subsequent coupling process, thus disrupting lignin formation^24^. Based on this observation, we engineered a set of novel *O*-methyltransferase (OMT) variants using structure-guided iterative saturation mutagenesis. These OMT variants effectively catalyse the 4-*O*-methylation of monolignols, and are therefore termed monolignol 4-*O*-methyltransferases (MOMTs)^24^ (Fig. 1c). One of these variants, MOMT4, shows a slight catalytic preference for methylating sinapyl alcohol, the S-lignin precursor, *in vitro*^25^. To evaluate its potential effects on lignin synthesis in woody species, and its application in tailoring wood digestibility for biofuel production, in this study we express *MOMT4* in hybrid aspen (*Populus tremula* × *Populus alba*), a fast growing *Populus* species. We demonstrate that the expression of *MOMT4* in aspen markedly alters lignin structure, with more than a 50% reduction of the labile S-units and about a fourfold increase in condensed lignin subunit cross-linkages by G-units. However, in contrast to our common perception, along with the increase of condensed lignin structures, the efficiency of the release of simple sugars from transgenic woods increases up to 62%, and the ethanol yield from pretreated and non-treated transgenic woods rise by >40% and 49%, respectively, compared with those from their corresponding controls. Furthermore, the content and composition changes in lignin do not affect growth and fitness of the transgenic aspens in the greenhouse; the wood densities and the biomass yields are comparable to those of the controls. These data suggest that (1) lignin condensation itself is not a critical factor affecting the digestibility of the cell wall; (2) the developed strategy of etherifying lignin precursors by the engineered OMT represents a useful biotechnological solution for effectively tailoring the digestibility of *Populus* woody biomass and, (3) the created transgenic aspens are a promising advanced biomass feedstock for biofuel production.

## Results

### Alteration of lignin content and structure

**Figure 2:**
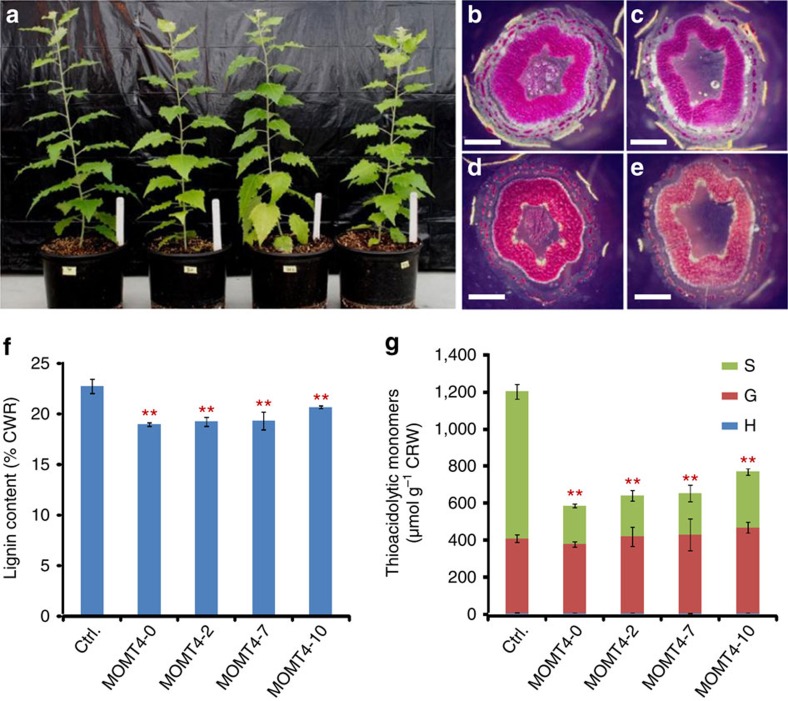
The effect of expression of *MOMT4* on content and composition of lignin in transgenic aspens. (**a**) Three-month-old hybrid aspens of control (left) and three *MOMT4* independent transgenic lines (right). (**b**,**c**) Phloroglucinol-HCl staining of the stem cross-sections of control (**b**) and *MOMT4-0* transgenic line (**c**). (**d**,**e**) Mäule staining of the stem cross-sections of control (**d**) and *MOMT4-0* transgenics (**e**). Scale bars, 1 mm. (**f**) Acetyl bromide total lignin content in the cell walls of control and *MOMT4* transgenic aspen stems. (**g**) The monomers released by thioacidolysis from the stem cell walls of *MOMT4* transgenic aspens; S, syringyl; G, guaiacyl; H, *p*-hydroxyphenyl; CWR, cell wall residues; Ctrl., control. Data in **f**,**g** represent mean±s.e. with three biological replicates (each with three technical repeats) for the control and three technical repeats for the individual transgenic lines. ** Indicates significant difference of lignin content (**f**) or S-monomer (**g**) compared to the control with *P*<0.01 (Student's *t*-test).

MOMT4, one of engineered enzyme variants from the parent enzyme isoeugenol 4-*O*-methyltransferase^26^, possesses four amino acid substitutions in its active site, that is, T133L, E165I, F175I and H169F (ref. 25). Those substitutions confer a substantial catalytic efficiency of the enzyme to both coniferyl alcohol and sinapyl alcohol for *p*-hydroxyl methylation. Kinetically, MOMT4 slightly prefers sinapyl alcohol with a catalytic efficiency (*k*_cat_/*K*_m_) of 3,999.3 (M^−1^ s^−1^), and a binding affinity (*K*_m_) of 68.1±11.1 (± indicates s.d., *n*=3) μM versus 2,738.7 (M^−1^ s^−1^) and 192.6±24.5 (± indicates s.d., *n*=3) μM for coniferyl alcohol. We transferred the expression cassette of the *MOMT4* gene, driven by the promoter of the bean phenylalanine ammonium lyase-2 (*PAL2*) gene that encodes the enzyme catalysing the first step of phenylpropanoid/lignin biosynthesis^27^, into hybrid aspen via *Agrobacterium*-mediated transformation (Fig. 2a). Bean *PAL2* promoter has been demonstrated to be highly active in the early stage of vascular development at the inception of xylem differentiation, when it drives the glucuronidase reporter gene, and is heterologously expressed in tobacco^28^; its promoter property was well conserved, even expressing in gymnosperm species^29^. Furthermore, this promoter has been successfully adopted in driving the target genes to effectively disturb lignin biosynthesis in different plants^14^,^25^. After obtaining the independent primary transformants, ∼2-month-old transgenic trees grown in a greenhouse were initially screened. Profiling the methanolic extracts from the plants revealed that two novel metabolites had accumulated in the leaves of the established transgenic lines (Supplementary Fig. 1a,b). After digestion of the leaf extract with β-glucosidase, we found that one metabolite we resolved was identical to the 4-*O*-methylated coniferyl alcohol, and the other one was identical to the 4-*O*-methylated sinapyl alcohol, based on their retention times and ultraviolet spectra, compared with the enzymatically synthesized authentic standards, as was their molecular mass assignment (detected by gas chromatography–mass spectrometry (GC–MS)) (Supplementary Fig. 1a–f). These data indicate that the engineered MOMT4 functions properly in the transgenic aspens, and can modify monolignols into the corresponding 4-*O*-methylated compounds; those methylated products most likely were further transformed to their glucoconjugates for detoxification and/or for the storage. The levels of the accumulated metabolites in the leaves of ∼2-month-old trees were ∼2–3 μmol g^−1^ of the fresh weight of the leaves for the 4-*O*-methylated coniferyl alcohol, and 5–7 μmol g^−1^ fresh weigh for the 4-*O*-methylated sinapyl alcohol (Supplementary Fig. 1g). Interestingly, in the stems of ∼2-month-old trees, only 4-*O*-methylated sinapyl alcohol was detected, and its accumulation level was much lower than that in the leaves at ∼0.12–0.2 μmol g^−1^fresh weight, which probably reflects the limited storage capacity of xylem cells for the soluble phenolics. Based on the phenolic profiling and the preliminary quantification of the total lignin content of these generated transgenic lines, we selected four independent lines, covering the lowest- to the highest-level of metabolic changes, for our further detailed histochemical and chemical analyses on the composition of their cell walls. The conventional phloroglucinol-HCl stain that yields a violet-red colour indicative of total lignin revealed that the xylem tissues of *MOMT4* transgenic aspens exhibited a slightly weaker (or nearly indiscernible change in) colour intensity than those of the control plants (Fig. 2b,c), suggesting a limited reduction of total lignin content in the wood; however, when we applied the Mäule stain (which stains syringyl units in lignin red), the colour intensity apparent on the cross-section of *MOMT4* transgenic stem was undoubtedly fainter than that displayed in the control stem (Fig. 2d,e), indicating a substantial reduction of S-type lignin subunits in the cell walls of *MOMT4* transgenic lines. Consistent with the histochemical observation, quantification of aceteyl bromide lignin in the stem cell walls of ∼6-month-old plants revealed that the average lignin content of control lines was ∼23% of tissue dry weight, whereas of the *MOMT4* transgenic lines ranged from 19 to 20%, indicating a moderate level of lignin reduction (Fig. 2f). When the monomeric composition of lignin was determined using the diagnostic thioacidolysis method that primarily cleaves β–*O*–4-ether linkages interconnecting the lignin subunits^30^, we found that the amount of released S monomers from *MOMT4* overexpression lines exhibited 63–74% reduction compared with the control lines; in contrast, the amount of released G-units was very similar, with an approximate level of 400 μmol g^−1^ dried cell wall residues (Fig. 2g). Correspondingly, the S/G ratio of lignin had fallen from a value of 2:1 in the controls to 1:2 in the *MOMT4* overexpression lines (Fig. 2g). These data suggest that expression of *MOMT4* in hybrid aspen substantially alters lignin composition, and that the incorporation of the S-lignin monomer (sinapyl alcohol) was principally impaired in the cell walls of *MOMT4* transgenics.

**Figure 3:**
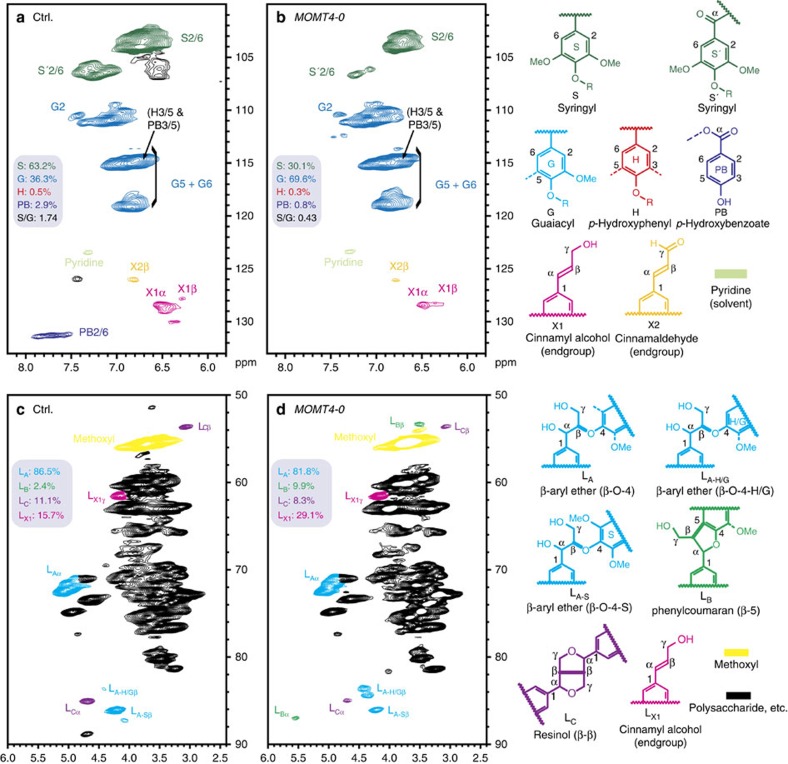
2D HSQC NMR spectral analysis of lignin structure in *MOMT4* transgenic aspens. (**a**,**b**) Partial short-range 2D HSQC NMR spectra (aromatic region) of equal amounts of solubilized total cell walls of control and *MOMT4* transgenic plants. The main structural units are coloured to coincide with their structures on the right. (**c**,**d**) Partial short-range 2D HSQC NMR spectra of lignin aliphatic and polysaccharide region of the same samples. The main units, characterized by their inter-unit linkages, are coloured to coincide with their structures to the right. See Supplementary Figs 2 and 3 online for the complete set of spectra of the transgenic and control samples.

Two-dimensional heteronuclear single-quantum coherence (2D HSQC) NMR experiments further verified the reduction of S-units in lignins from *MOMT4* transgenic lines. The detected signals from the S-units of two independent *MOMT4* transgenic lines exhibited >50% depletion over than those of the control lines; the calculated S/G ratio changed from 1.7 in the control lines to 0.4 in the *MOMT4* overexpression aspens (Fig. 3a,b; Supplementary Fig. 2), consistent with the thioacidolysis results. In both the thioacidolytic and NMR analyses, the detected amount of the *p*-hydroxyphenyl (H) units was extremely low, and was not significantly different between the *MOMT4* overexpression and control lines (Figs 2 and 3).

**Figure 4:**
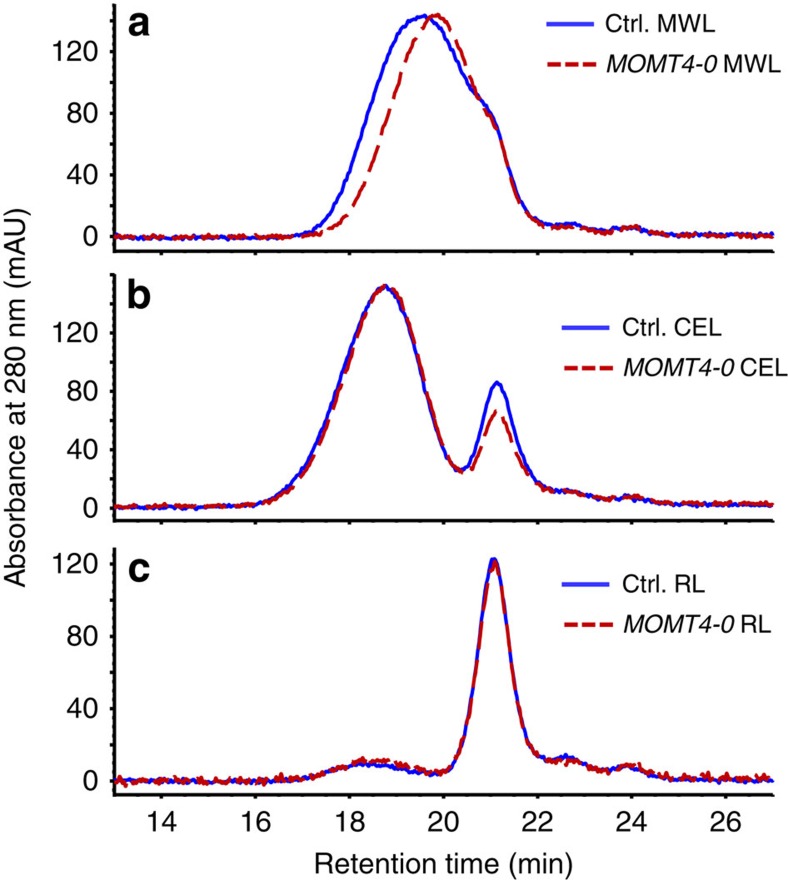
Molecular weight profiles of different lignin fractions from the cell walls of *MOMT4* transgenic and control plants. (**a**) The lignins extracted from the ball-milled woods of control and *MOMT4-0* transgenic plants (referred to as MWL). (**b**) The lignins extracted from remaining materials after extraction of MWL, followed by a cellulolytic enzyme digestion (termed as CEL). (**c**) The lignins extracted from remaining wood residuals after extraction of MWL and CEL (referred to as RL). The prepared lignins were further acetylated and then analysed by HPLC.

The aliphatic region of 2D HSQC NMR spectra reflects the changes in the types and distribution of inter-unit linkages present in the lignin fraction. The lignin from the control lines was rich in β-aryl ether units with modest amounts of resinols and low amounts of phenylcoumarans, as well as modest levels of the cinnamyl alcohol end groups (Fig. 3c; Supplementary Fig. 3). Resinols (β–β-coupled units) mostly arise from the dimerization of sinapyl alcohol. The spectra of lignin derived from *MOMT4* transgenic aspens revealed that the resinol and β-aryl ether units were decreased by 25% compared with the controls, consistent with the lower amount of syringyl monomers released by thioacidolysis and documented in 2D HSQC NMR spectra. However, the phenylcoumaran (β–5-coupled) structures that require at least one guaiacyl unit for their formation increased on average by more than fourfold over the controls (Fig. 3d; Supplementary Fig. 3). The level of cinnamyl alcohol end groups that also primarily arise from coniferyl alcohol (G-unit) monomer–monomer coupling^3^, rose about 1.5-fold (Fig. 3d; Supplementary Fig. 3). These data affirm that lignin polymer from *MOMT4* transgenic aspens is richer in the condensed linkages (for example, β–5 structures), and may be more branched or simply have shorter chains, as hinted by its increased end group units. Indeed, gel permeation chromatography revealed that the distribution of molecule weight (*M*_r_) in the milled wood lignin fraction is lower in the *MOMT4* transgenics compared with the control samples (Fig. 4), suggesting a lower degree of polymerization.

### Alteration of wall-bound phenolics

**Figure 5:**
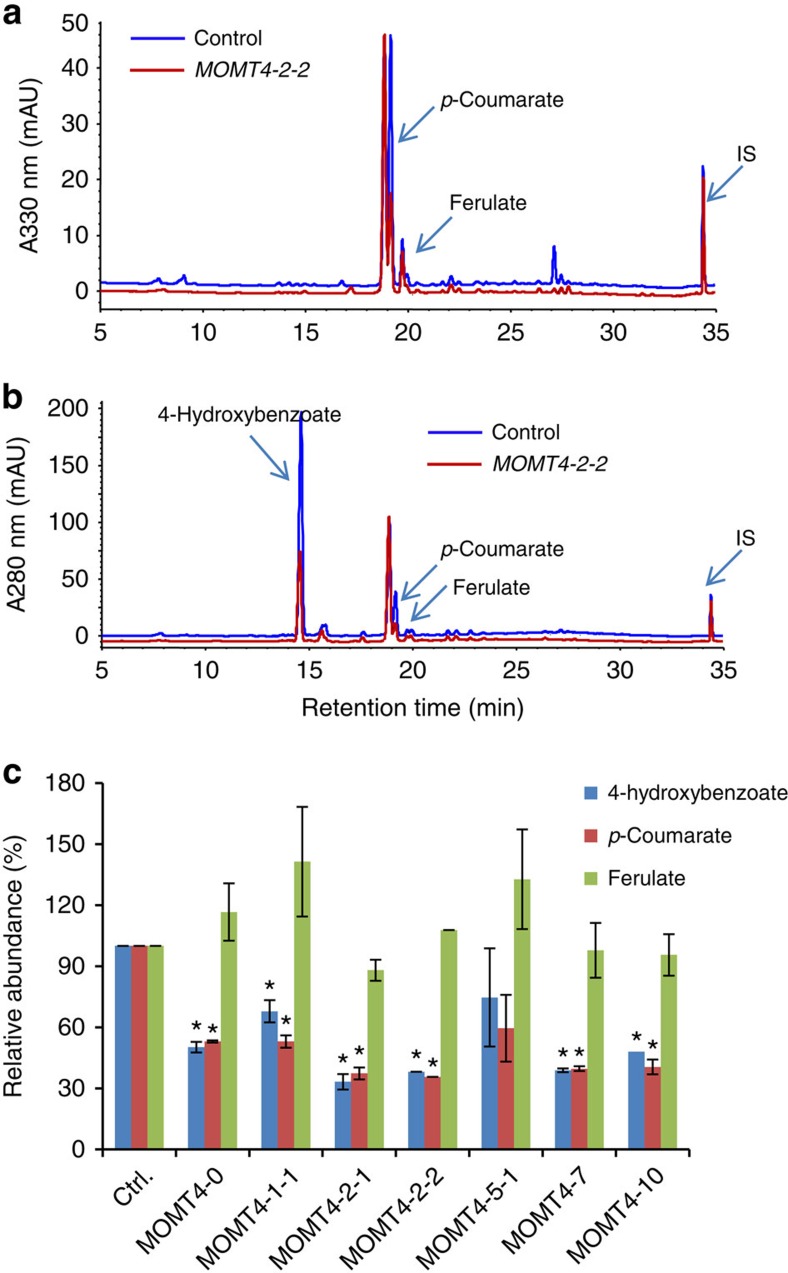
Alteration of the accumulated wall-bound phenolics in stem cell walls of *MOMT4* transgenic aspens. (**a**,**b**) UV-HPLC profiles of wall-bound phenolic extract from *MOMT4-2-2* stem cell walls with ultraviolet absorbance at 330 nm (**a**) and 280 nm (**b**), respectively, to optimally display different phenolics; IS, internal standard. (**c**) Calculated relative abundance of the detected wall-bound phenolics in the individual *MOMT4* transgenic lines. The averaged amount detected in the control lines was set as 100%. Data were from five biological replicates (each with two technical repeats) for the control set and two to three technical repeats for the individual transgenic lines. Error bar stands for s.d. *Indicates significant difference compared to the corresponding controls with *P*≤0.05 (Student's *t*-test).

Hydroxycinnamates (for example, *p*-coumarate and ferulate) and hydroxybenzoates that bond with the cell walls' non-cellulosic polysaccharides and lignin via ester linkages can act as crosslinkers or play a role in altering the oxidative potential of monolignols^31^. In poplar cell walls, the prominent ‘wall-bound' phenolic is *p*-hydroxybenzoate that predominantly acylates syringyl units^32^,^33^. Examining the wall-bound phenolics from the stems of *MOMT4* transgenics revealed that the levels of both *p*-hydroxybenzoate and *p*-coumarate showed about a 50% reduction, whereas the average amount of ferulate essentially was unchanged, compared with those in the control lines (Fig. 5). Both *p*-hydroxybenzoate and *p*-coumarate were proposed to conjugate with sinapyl alcohol within the cell and then incorporate into lignin polymer^31^. The reduction of both *p*-hydroxybenzoate and *p*-coumarate esters was consistent with the decreased incorporation of S-lignin monomers.

### Alteration of cell wall polysaccharides

**Figure 6:**
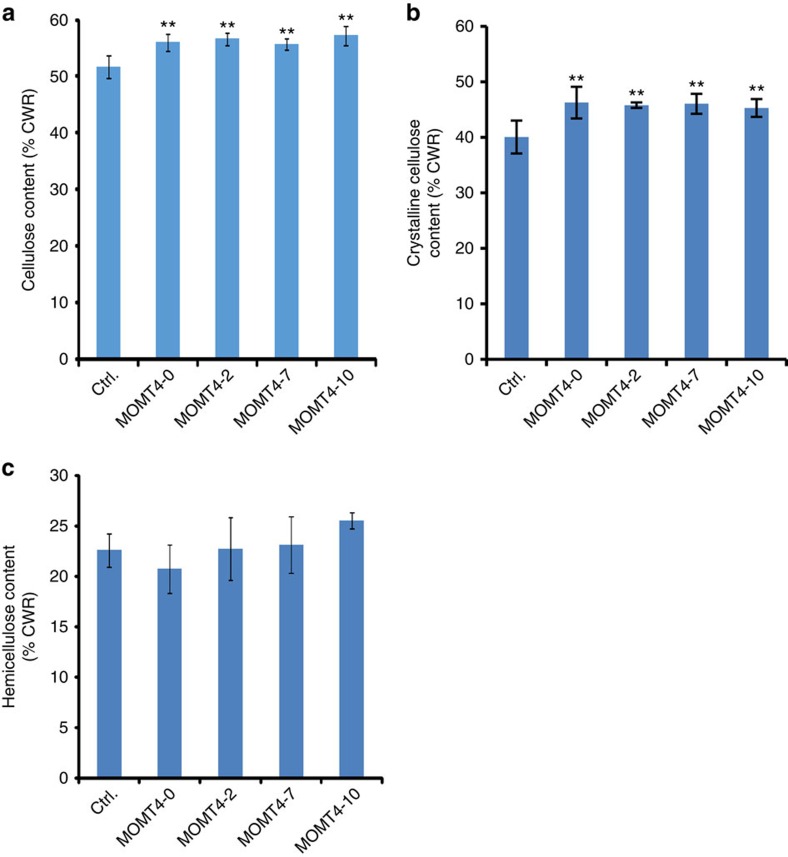
The effect of expression of *MOMT4* on accumulation of carbohydrates in transgenic aspens. (**a**) Cellulose content in the cell walls of control and *MOMT4* transgenic aspens. (**b**) The crystalline cellulose content in *MOMT4* transgenic cell walls. (**c**) Total hemicelluloses content in the cell walls of control and *MOMT4* transgenic aspens. CWR, cell wall residues; Ctrl., Control. Data in **a**,**b** represent mean±s.d. from three biological replicates (each with eight technical repeats) for the control set and eight technical repeats for *MOMT4* OE lines. **P<0.01 (Student's *t*-test). Data in **c** represent mean±s.e. with three biological replicates (each with three technical repeats) for the control and three technical repeats for transgenic lines. No statistic difference between the transgenic and the control line was detected.

Along with changes of lignin content and structure of *MOMT4* transgenic aspens, the total cellulose content was found to have increased from 51% of the dried cell wall residues in the controls, to a maximum of 57% in the *MOMT4* overexpression lines, that is, by up to a 12% increase (Fig. 6a). Synchrotron FT-IR imaging further verified that the polysaccharide level in the developing xylem of the stem cross-section of *MOMT4* transgenics was substantially higher than that of the controls, whereas the level of lignification in the same developing wood tissues was lower (Supplementary Fig. 4). Along with the increase in total cellulose content, the levels of crystalline cellulose in the walls of *MOMT4* transgenics was also increased by up to 15% over the levels of the controls (Fig. 6b), suggesting an ultrastructural change of cellulose fibres. When hemicelluloses were quantified, we found that their abundance in the cell walls was essentially unchanged, accounting for about 22.6% of the cell wall dry mass in the control, and an average 23.2% (range 20.7–25.5%) in the transgenic lines (Fig. 6c). The detected amount of hemicellulosic monomeric sugars also remained essentially the same in most lines, except the glucose content, which may have arisen from the amorphous regions of cellulose fibres, which was slightly higher in one of the transgenics (Supplementary Table 1). These data demonstrate that the alteration of lignin content and structure in *MOMT4*-overexpressing aspens affects the accumulation and ultrastructure of cellulose fibres, whereas it has less effect on the deposition of hemicelluloses.

### Enhanced cellulolytic efficiency and ethanol yield

**Figure 7:**
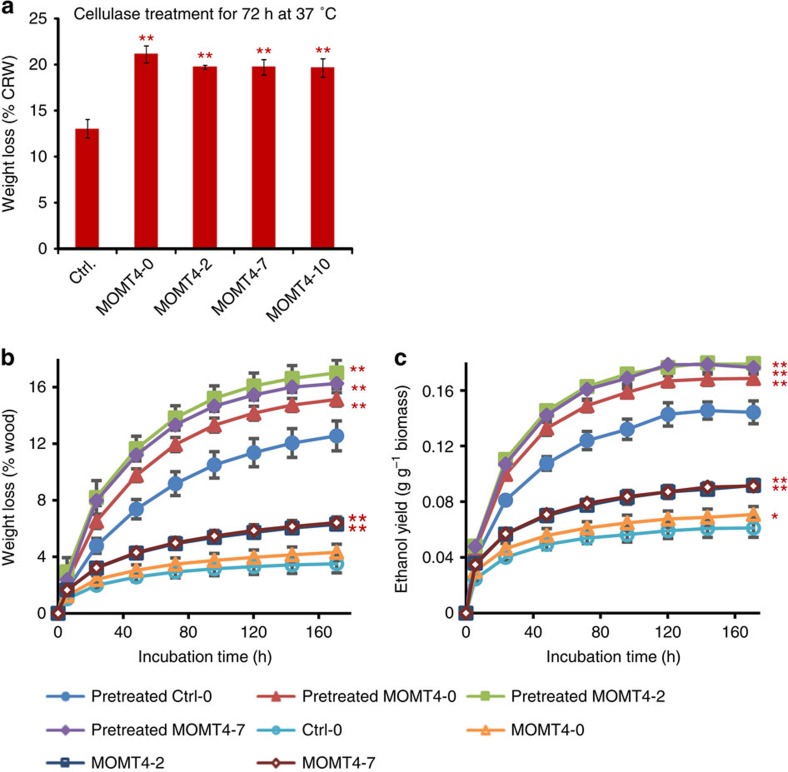
Enzymatic hydrolysis and bioconversion of *MOMT4* transgenic woods to ethanol. (**a**) Digestive weight loss when the prepared wood cell walls were treated with 2 mg ml^−1^ of cellulase for 72 h at 37 °C. Data represent mean±s.e. with three biological replicates (each with two to three technical repeats) for the control and three technical repeats for transgenic lines. CWR, Cell wall residues. **P<0.01 (Student's *t*-test). (**b**,**c**) Broth weight loss (Fermentation gas release) (**b**) and ethanol yield (**c**) during simultaneous saccharification and fermentation of the pretreated and untreated control and *MOMT4* transgenic woods. Data represent mean±s.d. of three biological replicates (each replicate with two experimental repeats for each control and transgenic lines. *P<0.05, **P<0.01 (Student's *t*-test).

When the prepared wood cell walls were treated with a cellulase mixture, all of the examined *MOMT4* transgenic samples displayed 50%–62% more weight loss than the controls, indicative of the release of more simple sugars and a higher degree of digestibility (Fig. 7a). Subsequently, two sets of the transgenic and control woods, one from 6-month-old newly propagated plantlets and another from ∼1-year-old basal stem grown from the trees after three coppice events, underwent ‘simultaneous saccharification and fermentation'^6^ to measure ethanol production. Both the *MOMT4* transgenic woods and control ones either were treated with alkali (Supplementary Fig. 5a) or left untreated; then after washing the pretreated samples with water, the woody powders were incubated with polysaccharidase enzymes and yeast cells in the fermentation broth. During incubation, we measured the weight loss caused by the release of fermentation gases and the ethanol content in the broth. The data showed that both the pretreated and non-pretreated transgenic woods displayed both a faster and higher fermentative weight loss (that is, the gas release), indicating a higher rate of saccharification and bioconversion compared to the control woods (Fig. 7b; Supplementary Fig. 5b). Correspondingly, higher yields of ethanol were realized compared to their respective controls (Fig. 7c; Supplementary Fig. 5c). When simultaneous saccharification and fermentation proceeded for 168 h, the alkaline-pretreated and non-pretreated wood samples from 6-month-old *MOMT4* transgenic plantlets showed up to 24% and 49% increase in ethanol yield, respectively (Fig. 7c). For alkaline-pretreated and non-pretreated wood samples from ∼1-year-old basal stem of the primary transgenic plants, ethanol yield increased up to 40% and 48%, respectively, compared with the controls (Supplementary Fig. 5c). These data indicate that even though the *MOMT4* transgenic woods contain a relatively higher level of condensed lignin, their digestibilities and bioconversion rates were substantially improved.

### Growth and wood anatomy of transgenic aspens

**Figure 8:**
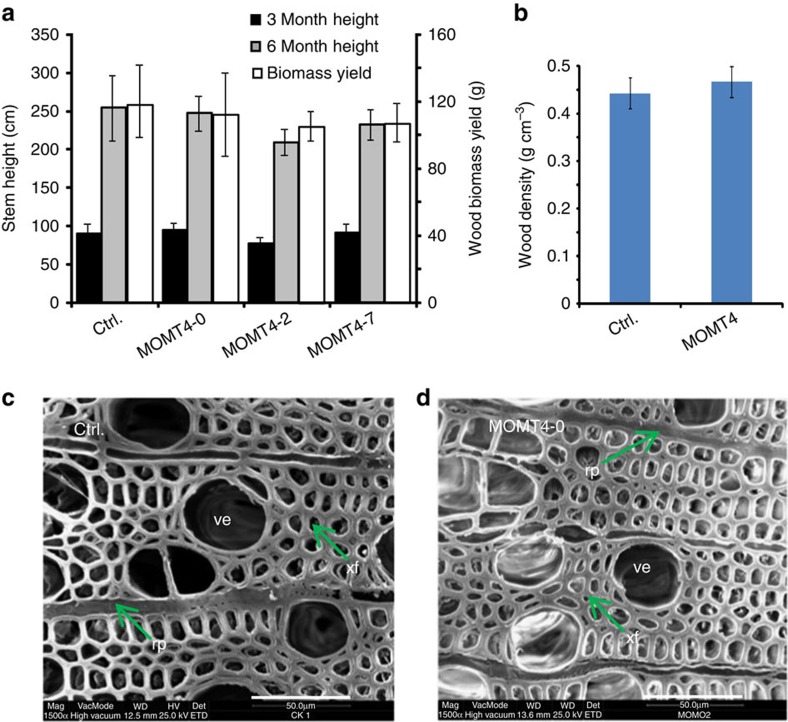
Wood biomass yield, density and anatomy of *MOMT4* transgenic aspen. (**a**) The measured stem height and dried wood biomass yield of 3- and/or 6-month-old plants of control and *MOMT4* transgenics grown in a greenhouse. Data are mean±s.d. with three biological repeats for control and each transgenic line. (**b**) The calculated average wood density of the basal stems of ∼8-month-old aspens. Data are mean±s.d. with six and eleven biological replicates (each replicate with three technical repeats) for control and transgenic plants, respectively. (**c**,**d**) Scanning electron micrographs of stem transverse section of ∼2-month-old control (**c**) and *MOMT4-0* transgenic line (**d**). rp, ray parenchyma; ve, vessel element; xf, xylary fibre. Scale bar, 50 μm.

Although the *MOMT4* transgenic aspens exhibited substantial changes in both lignin and cellulose contents and structures, they exhibited normal growth and development in the greenhouse (Fig. 8). The average stem heights, basal stem thicknesses and total dried biomass yields of 3- and/or 6-month-old transgenic trees were statistically similar to those of the controls, notwithstanding the substantial variation among individual transgenic and control plants (Fig. 8a; Supplementary Table 2). The measured wood density of a set of primary transgenic lines, on average, was the same as in the control lines (Fig. 8b). At the cellular level, *MOMT4* transgenic aspens developed a normal vasculature, and no collapsed vessel or fibre cells was observed, except that the lumina of their fibres appeared slightly narrower than those of the control line (Fig. 8c,d). These data suggest that expression of *MOMT4* and the alteration of the composition and structure of lignin do not significantly compromise the aspen trees' growth or their production of woody biomass.

### Syringyl lignin related gene expression and enzyme activity

Sinapyl alcohol biosynthesis branches from coniferyl alcohol/aldehyde biosynthesis in the pathway by the sequential catalyses of coniferaldehyde/ferulate 5-hydroxylase (Cald5H/F5H) and COMT. We hypothesized that the reduced S-lignin content in the transgenics might have resulted from the potential inhibitory effect of 4-*O*-methylated monolignols, the products of MOMT4, on the activities of the biosynthetic enzymes specific for syringyl monomers. We therefore conducted substrate-inhibition assays using the prepared 4-*O*-methylated coniferyl and sinapyl alcohols on the recombinant aspen enzymes, Cald5H/F5H, COMT, CAD ((hydroxy)cinnamyl alcohol dehydrogenase), and a homologue of the previously identified poplar SAD (sinapyl alcohol dehydrogenase)^34^, as well as on the crude enzyme extracts (for COMT, CAD/SAD, and peroxidase activities). Except for the recombinant CAD and the SAD homologue^34^, where their activities showed a 3–14% inhibition when incubated with 4-*O*-methylated monolignols (Supplementary Table 3), there was no obvious inhibition on the activities of other recombinant enzymes (Supplementary Table 3). Consistent with a previous study^24^, the 4-*O*-methylated monolignols did not impair the peroxidase-mediated oxidative polymerization process of the conventional monolignols (Supplementary Fig. 6).

Considering the potential global transcriptional effect of the expression of *MOMT4* on the cell wall and the lignin biosynthetic genes, we conducted a transcriptomic analysis on an *MOMT4* overexpression line using RNA-seq. The data indicated that although the transcripts of *MOMT4* transgene were highly abundant, which verifies the overexpression of the *MOMT4* gene and demonstrates the reliability of RNA-seq dataset, none of the lignin biosynthetic genes exhibited substantial changes in their transcript abundances compared with those of the control set. Quantitative RT-PCR analysis on a set of xylem-specific lignin biosynthetic genes^35^ further validated that the transcripts of monolignol-branch genes, particularly the S-lignin synthetic genes, *Cald5H*/*F5H*, *COMT*, *CAD* and *SAD* homologue, did not exhibit significant differences between *MOMT4* transgenic and control lines, although transcripts of a few genes in individual control or *MOMT4* transgenic lines appeared to be outliers (Supplementary Fig. 7). Interestingly, RNA-seq data indicated that, among a small set of genes showing an altered transcript abundance, a few are annotated to encode cell wall proteins, including an α-expansin family of proteins that are potentially involved in cell wall loosening^36^, a glycosyl hydrolase (9B13) whose homologous members in the family of *endo*-1,4-β-glucanases were reported to participate in cellulose synthesis or assembly^37^, and the cell wall-localized proline-rich protein (Supplementary Table 4). These data suggest the potential involvement of those proteins/enzymes in the observed alteration of the cell wall structure and composition of the *MOMT4* transgenic aspens.

## Discussion

The production of biofuels and bio-based chemicals from woody biomass is limited by its recalcitrance to hydrolysis for releasing simple sugars. Most lignin elimination processes are inefficient and expensive for many applications. Reducing and/or structurally changing lignin by manipulating certain genes in the monolignol biosynthetic pathway can enhance the cell wall degradability^1^; however, this approach is often accompanied by impairment of plant growth and development that results in a severe penalty to biomass yield^6^,^12^,^13^,^17^,^18^. Plants seem, however, to be tolerant of a wide range of lignin compositional/structural changes. One measure of lignin structure is its G- and S-lignin unit composition. In general, a high S/G ratio is associated with increased pulping yields^38^,^39^, or improved enzymatic release of sugars^40^. The present study demonstrated that expression of the MOMT4 enzyme that etherifies the phenolic moieties of monolignols preferentially disrupts S-lignin deposition in hybrid aspen, which leads to the production of a G-lignin-rich biopolymer. An increase in G-units would be expected to exacerbate lignin's condensation, complexity and thus recalcitrance of the cell walls. Surprisingly, the *MOMT4* transgenics' woods displayed substantially improved efficiency in sugar release and had an enhanced bioconversion rate to ethanol. Up to 60% more woody biomass could be digested from transgenic cell walls than from the control wood under the same treatment, and up to 49% more ethanol resulted from the conversion of the non-pretreated transgenic woods, in comparison with the controls (Fig. 7; Supplementary Fig. 5). These results suggest that expression of *MOMT4* in hybrid aspen leads to a significant alleviation of the recalcitrance to wood processing in an unexpected way, facilitating the conversion of woody biomass to liquid fuels. The improved wood digestibility of *MOMT4* transgenic aspen may be attributed to several concurrent factors. For example, (1) the modest reduction of total lignin content in the cell wall that mitigates its physical barrier properties; (2) drastic structural alteration in lignin that may affect the association of lignin with polysaccharides and the interaction between cell wall biopolymers, thus changing the accessibility of the wall to digestive enzymes; (3) the relative increase of the content of cellulose fibres due to the cell wall's mass balance, which, in turn, enhances the source of fermentable six carbon sugars. Interestingly, altering lignin content and structure not only promoted cellulose deposition but also altered its ultrastructure; more crystalline cellulose microfibrils were formed in the *MOMT4* transgenics' cell walls (Fig. 6). The underlying mechanisms for such concomitant alteration in polysaccharide deposition and structure remain unclear. One hypothesis is that S-lignin subunits and/or their linked hydroxybenzoate esters may play structural roles in the lignin–polysaccharide association. Disturbing S-lignin synthesis may trigger a potential feedback remodelling of cell wall carbohydrates. Alternatively, S-lignin deposition is regarded as one of the integral steps of plant secondary cell wall formation^41^; disrupting S-lignin synthesis may disturb or delay the cell wall's development processes, sustaining wood at a younger stage, and thus rendering it less recalcitrant to breakdown. Collectively, our study suggests that lignin condensation does not necessarily correlate to the digestibility of *Populus*, and it is not the sole or critical factor dominating cell wall recalcitrance.

Expression of *MOMT4* in the herbaceous dicot *Arabidopsis*, and the woody species, aspen, yields quite different metabolic and biological consequences. When *MOMT4* expressed in *Arabidopsis*, the plants produced both the 4-*O*-methylated feruloyl and sinapoyl malates in their soluble phenolic fraction^25^, suggesting that the 4-*O*-methylated products of MOMT4 entered the endogenous pathway of phenolic-ester biosynthesis that is specific for *Brassicaceae* species^42^,^43^. The total lignin content in the stem of transgenic *Arabidopsis* exhibited a significant reduction, while its composition and structure essentially remained unchanged^25^. In contrast, when *MOMT4* was expressed in aspen, the transgenic plant accumulated glucoconjugates of the 4-*O*-methyalted monolignols (Supplementary Fig. 1); furthermore, the lignin from transgenic tree was composed of a significantly lower level of S-subunits, and more condensed (C–C bonded) structures containing G-subunits (Figs 2 and 3) than occurred in the control aspen. This biological discrepancy may reflect the diversity and speciation of the phenylpropanoid metabolism, and the detoxification mechanism that the particular plants adopt, and also the species variability of plant lignifications. In particular, in terms of the distinct effects of MOMT4 on lignin synthesis in these two species, *Populus* hardwood is rich in syringyl lignin with an S/G ratio ranging from 1.8 to 2.3^44^; whereas the lignin of *Arabidopsis* is guaiacyl rich (with S/G ratio ∼0.25)^45^. Since MOMT4 displays a discernible, yet less dominant, substrate preference to sinapyl alcohol, when it is expressed in the guaiacyl-rich *Arabidopsis*, its kinetic effect may be masked or compromised. Whereas when MOMT4 acts in an environment in which the S-monomeric substrate is more prominently available, its kinetic propensities may result in its dominantly modifying sinapyl alcohol, and thus triggering a metabolic consequence entailing a more severe disruption of S-lignin formation. Such metabolic preference in fact was also evidenced with the higher level of accumulation of the 4-*O*-methylated sinapyl alcohol than the 4-*O*-methyalted coniferyl alcohol in the soluble phenolic fraction of transgenic aspen leaves and stems (Supplementary Fig. 1). Another possibility for preferentially reducing S-lignin in the transgenic aspens is that the MOMT4-produced 4-*O*-methylated compounds may exert inhibitory effects on the activities of *Populus* intrinsic S-lignin-specific enzymes. However, our *in vitro* study revealed that the 4-*O*-methylated monolignols have no detectable inhibitory effects on the activities of Cald5H/F5H or COMT from aspen; they only displayed a 3–14% inhibition on the activities of cinnamyl alcohol dehydrogenases (Supplementary Table 3). With such an inhibition, both the activities of CAD/SAD to the G- and S- lignin precursors should be affected, which do not explain the observed preferential impairment of S-lignin synthesis (Supplementary Table 3). The exact reasons for expressing *MOMT4* in aspen preferentially to disrupt S-lignin formation may be complicated. It remains to be determined whether this phenomenon is *Populus* specific or occurs in the other woody species.

Despite the drastic change in lignin and cell wall structure, those greenhouse-grown, ∼6-month-old *MOMT4* transgenic aspens showed no penalty in biomass yield, nor loss in wood density. The structural integrity at the whole plant- and cellular-level was maintained essentially similar to that of the control (Fig. 8). The sustained growth and fitness features of *MOMT4* transgenic aspens are most likely attributed to their attaining condensed G-lignin, the increased cellulose content, and the elevated crystallinity of cellulose fibres in their cell walls along with a concomitant decrease in S-lignin formation. We note that the current study, examining the plant growth property, was conducted in a relatively short period. An extended physiological investigation, or a field trial with the generated transgenic trees, will substantiate their viability as biofuel feedstocks.

In summary, expressing an engineered monolignol 4-*O*-methyltransferase in hybrid aspen substantially alters lignin structure while also producing more cellulose. Such alteration facilitates wood digestibility and its bioconversion to ethanol, proving a useful strategy to tailor woody biomass for bio-based applications. The created transgenic aspen represents a valuable feedstock for further industrial exploitation in biofuel production.

## Methods

### Overexpression of *MOMT4* in hybrid aspen

Approximately 1.1 kb of the promoter of *PAL2* from bean (*Phaseolus vulgaris* L.) was amplified from the vector pCAMBIA2200-GW (ref. 14) with a pair of primers that incorporated HindIII and KpnI restrictive enzyme sites, respectively (Supplementary Table 5). The 35S promoter of the vector pMDC32, a gateway destination vector^46^, was removed by the enzymatic digestion with HindIII and KpnI and replaced with bean *PAL2* promoter; this yielded a binary vector pMDC32-*PAL*-GW. The open reading frame of the engineered *MOMT4* gene was then integrated into the created binary vector following the standard gateway cloning procedures (Invitrogene). The resulting *MOMT4* expression construct was transferred into *Agrobacterium tumefaciens* strain GV3101 for plant transformation. Hybrid aspen clone INRA 717-IB4 (*P. tremula* x *P. alba*) was propagated and transformed using the protocol of Ma *et al.*^47^ Briefly, leaf disc and stem segment explants, after wounding with multiple fine cuts, were co-cultivated for 1 h with *A. tumefaciens* strain GV3101 containing the *MOMT4* expression construct. Then, the explants were cultivated on callus induction medium (CIM, 0.025% (w/v) 2-(*N*-morpholino)ethanesulfonic acid (MES), 0.01% (w/v) *myo*-inositol, 0.43% (w/v) ½ MS basal salts, 3% sucrose, 10 μM 1-naphthaleneacetic acid, 5 μM 2-isopentyladenine, pH 5.8) at 22 °C in dark for 2 days. After washing with distilled water, the explants were subsequently cultured in dark on CIM containing 10 mg l^−1^ hygromycin and 200 mg l^−1^ timentin for selecting the transformed calli. After 2–3 weeks, the explants were sub-cultured on shoot selection medium (0.025% (w/v) MES, 0.01% (w/v) *myo*-inositol, 0.43% (w/v) ½ MS basal salts, 3% sucrose, 1% (v/v) FV vitamins) containing 0.2 μM thidiazuron, 10 mg l^−1^ hygromycin and 200 mg l^−1^ timentin for 2–3 months and sub-cultured every 3–4 weeks. The explants with multiple shoots were then transferred onto the shoot selection medium supplemented with 0.1 μM 6-benzylaminopurine, 10 mg l^−1^ hygromycin and 200 mg l^−1^ timentin for shoot elongation. The regenerated shoots were further screened for hygromycin resistance and induced for roots on ½ MS medium supplemented with 0.5 μM indole-3-butyric acid, 10 mg l^−1^ hygromycin and 100 mg l^−1^ timentin. After ∼30 days, the elongated, rooted shoots were transferred into the soil.

The generated primary transgenic plants were examined by RT-PCR for the expression of the *MOMT4* transgene, phenolic profiling and preliminary total lignin content determination. The selected primary transgenic clones were then propagated. Briefly, the nodal and tip explants were taken from actively growing shoots of coppiced plantlets. After sterilizing them, they were placed in Yellow medium^47^ and sub-cultured frequently until a uniformly continuous growth of new shoots was obtained. The shoots then were harvested and rooted in the rooting medium^47^. The generated plantlets were transferred into the pots, and maintained in the greenhouse at 25 °C under high illumination at 500 μmol m^−2^ s^−1^. When the plants were fully established, they were then illuminated by natural light. The transgenic and control plants intended for lignin and phenolic analysis were grown side by side.

### Methanolic soluble phenolic profiling

Approximately 2-month-old plantlets of the control lines and the *MOMT4* transgenic lines were harvested. All the leaves on a shoot of about 30 cm (above ground), and the shoot itself (without bark) were collected from individual lines and ground into fine powders under liquid nitrogen. Approximately 0.2 g of the leaf or stem powders were extracted overnight with 1 ml 80% methanol (containing 20 μM chrysin as the internal standard). Then, 25 μl extracts of leaf, or 200 μl extracts of stems after solvent evaporation were digested with 2 mg ml^−1^ β-glucosidase in 500 μl citric-phosphate buffer (pH 7.0), and then re-extracted with ethyl acetate. After drying them under a steam of N_2_, the ethyl acetate extracts were re-dissolved in 100 μl methanol, and 30 μl was taken for HPLC analysis. For ultraviolet-HPLC profiling, the samples were resolved in a mobile phase of 0.2% acetic acid (A) with an increasing concentration gradient of acetonitrile containing 0.2% acetic acid (B) at 0–2 min, 5% (B); 2–30 min, 5 to 50% (B); 30–32 min, 50 to 100%, and then 100% for 2 min at a flow rate of 1 ml min^−1^. The ultraviolet absorption was monitored at 254-, 280-, 310- and 330-nm using a multiple-wavelength photodiode array detector. The detected compounds were quantified based on their peak areas and calculated against the standard curves of conifery alcohol and sinapyl alcohol as they exhibit nearly the same ultraviolet absorption property as do their 4-*O*-methylated compounds.

The 4-*O*-methylated coniferyl alcohol and sinapyl alcohol used for identifying the leaf metabolites were synthesized by the MOMT4-catalysed reactions as described below in the section of Enzyme activity inhibition assay.

For characterizing the novel metabolites by GC–MS, the leaf extracts after β-glucosidase digestion were re-extracted with ethyl acetate. After drying under N_2_ gas, the residuals from the extracts were derivatized with 30 μl pyridine and 30 μl MSTFA at room temperature for 4 h, and then dried under a stream of N2 gas. Dichloromethane (60 μl) were used to dissolve the samples. Around 1 μl was injected for GC–MS analysis. The mass spectra of two distinct metabolites in the chromatographs of transgenic samples were recorded.

### Histochemical analyses

Histochemical analyses was performed on 30-μm-thick sections taken from the same position of basal node of 2-month-old shoots of the control and *MOMT4* transgenic aspens. For phloroglucinol (Wiesner) staining, the fresh sections were left for 5 min in 1% phloroglucinol in 95% ethanol and mounted in 6 N HCl. For Mäule staining, the stem sections were immersed in 1% (w/v) potassium permanganate solution for 5 min at room temperature and then washed with water and acidified with 3% hydrochloric acid until partially decoloured. Sections were finally mounted in ammonia or 5% NaHCO_3_ and examined quickly. Photographs were taken by using a Leica stereomicroscope system with a Leica DFC 300 colour camera.

### Analysis of wall-bound phenolics and lignin

Aspen stems were harvested from 6-month-old plants. Segments of basal stems ∼30 cm long, taken from each of the transgenic and control lines, were peeled to remove bark, dried and then ground in a Wiley mill. The milled wood chips were passed through a 60-μm mesh sieve. The wood powders were then extracted with 70% ethanol at 65 °C for 3 h. This process was repeated for three times. The residuals were further extracted three times with chloroform/methanol (1:1, v/v), and then treated overnight with acetone at room temperature. The extracted free residuals were dried at 45 °C and then ball-milled into a fine powder. The wall-bound phenolics were extracted with 2 N NaOH in the dark at 37 °C for 16 h. After neutralization of the reaction with concentrated HCl, the hydrolysate was extracted with water-saturated ethyl acetate twice. The extract was dried under stream of N_2_ gas then dissolved in 100 μl 80% methanol and analysed using HPLC with the aforementioned method (in soluble phenolics profiling section).

Total lignin was quantified by the acetyl bromide method^48^. Extractive-free wood powders (5 mg) were incubated with 1 ml 25% acetyl bromide in acetic acid at 70 °C for 30 min. After cooled down and diluted with 5 ml acetic acid, 300 μl solution was aliquoted and neutralized with 400 μl 1.5 M NaOH and 300 μl 0.5 M hydroxylamine hydrochloride. The neutralized sample was further diluted with 1.5 ml acetic acid and the absorbance at 280 nm was measured. The extinction coefficient of 18.21 g^−1^ l cm^−1^ was used for the calculation of lignin content.

The thioacidolysis method^49^ was followed to estimate the monomeric composition of lignin. Briefly, for each sample, 10 mg of extractive-free wood powders were mixed with 1 ml of freshly prepared reaction mixture (2.5% boron trifluoride etherate and 10% ethanethiol in distilled dioxane (v/v)) in a 2 ml glass vial and flushed with N_2_ gas. Then the vial was tightly sealed and heated at 95 °C for 4 h with periodic agitation. The reaction was stopped by placing on ice for 15 min and then its pH value was brought to 3–4 by using 0.4 M sodium bicarbonate. The reaction solution was transferred to a new 10 ml vial; 2 ml water was added. Meanwhile, 1 mg tetracosane (dissolved in 1 ml methylene chloride as internal standard) was added to each vials. The vial was recapped, vortexed, then allowed to settle for more than half hour until phase separation of the solution occurred. An aliquot (1.5 ml) of the organic phase was taken and passed through a Pasteur pipette packed with an inch of anhydrous sodium sulfate. The filtrate was then evaporated to the dryness and resuspended in 0.5 ml of methylene chloride. Samples (50 μl) were then dried and derivatized with pyridine and *N*-methyl-*N*-(trimethylsilyl) trifluoroacetamide (Sigma) at room temperature for 5 h. Quantifying the corresponding monomers was accomplished via a gas chromatography-flame ionization detector on a GC instrument (Agilent 7890A) after an appropriate calibration relative to the tetracosane internal standard.

### Whole-cell wall NMR analyses

The pre-ground cell walls were extracted with distilled water (ultrasonication, 1 h, three times) and 80% ethanol (ultrasonication, 1 h, three times). Isolated cell walls were dried and ball-milled in a Planetary micro mill Pulverisette 7 premium line (Fritsch, Idar-Oberstein, Germany) at 600 r.p.m., using ZrO_2_ vessels (20 ml) containing ZrO_2_ ball bearings (10 × 10 mm). Each sample (180 mg) was ground for 1 h 10 min (interval: 10 min, break: 5 min, repeated nine times). The cell walls (60 mg) were collected directly into the NMR tubes, and gels were formed using DMSO-d_6_/pyridine-d_5_ (4:1, v/v, 0.5 ml) with sonication (30 min)^50^.

NMR spectra were acquired on a Bruker Biospin (Billerica, MA, USA) Avance 700 MHz spectrometer equipped with a cryogenically cooled 5-mm quadruple-resonance ^1^H/^31^P/^13^C/^15^N QCI gradient probe with inverse geometry (proton coils closest to the sample). The central DMSO solvent peak was used as an internal reference (*δ*_C_ 39.5, *δ*_H_ 2.49 p.p.m.). The ^1^H–^13^C correlation experiment was an adiabatic HSQC experiment (Bruker standard pulse sequence ‘hsqcetgpsisp2.2'; phase-sensitive gradient-edited-2D HSQC using adiabatic pulses for inversion and refocusing). HSQC experiments were carried out using the following parameters: acquired from 11.5 to −0.5 p.p.m. in F2 (^1^H) with 1,682 data points (acquisition time 100 ms), 215 to −5 p.p.m. in F1 (^13^C) with 620 increments (F1 acquisition time 8.0 ms) of 32 scans with a 0.5 s interscan delay (D1); the d_24_ delay was set to 0.86 ms (1/8 J, *J*=145 Hz). The total acquisition time for a sample was 5 h. Processing used typical matched Gaussian apodization (GB=0.001, LB=−0.5) in F2, and squared cosine-bell and one level of linear prediction (32 coefficients) in F1. Volume integration of contours in HSQC plots used Bruker's TopSpin 3.1 (Mac) software. Assignments of peaks from NMR spectra were based on previous publications^50^.

### Cellulose quantification and cellulolytic analysis

For cellulose content determination, the control and transgenic wood powders (passed through a 60-mesh sieve) were sequentially treated with 75% ethanol, chloroform/methanol mixture (1:1), and acetone as described above, then resuspended in 0.1 N sodium acetate (pH 5.0) and mixed with 5 μg amylase (Sigma) to each sample to incubate at 37 °C, overnight. After digestion, the recovered powders were washed with water and acetone, respectively, three times, the powders were then dried at 35 °C and further ground using a ball mill. Cellulose content was measured according to the Updegraff's method^51^. For determining crystalline cellulose content, we adopted the trifluoroacetic acid (TFA)-phenol-sulfuric assay^52^. Briefly, ∼1 mg extract-free cell wall residuals were mixed with 1 ml of 2 M trifluoroacetic acid and heated at 121 °C for 90 min, with applying a vortex every 30 min to break up the chunks. After centrifugation, we removed the supernatant representing non-cellulose monosaccharides and amorphous cellulosic sugars, and then the pellets were collected and washed. The pellets next were suspended in 100 μl of 50 mM sodium acetate buffer (pH 4.8) and 100 μl freshly made 5% phenol, and then briskly mixed with 1 ml concentrated sulfuric acid. The orange colour was developed at 30 °C in 2 h, and its intensity was quantified at 500 nm. Glucose was used as the authentic standard.

The susceptibility to cellulolytic breakdown was evaluated using a method described by Sibout^53^. Extract-free samples (200 mg) were placed in 30 ml of 50 mM sodium acetate buffer (pH 4.8), containing 2 mg ml^−1^ of Cellulase (Onozuka R-10, PhytoTechnology Laboratories), and incubated at 37 °C for 72 h under magnetic stirring. After incubation, the reaction medium was filtered over a filtering crucible. The residue then was washed with water, oven-dried and gravimetrically determined.

### Hemicelluloses quantification

The determination of the content of hemicelluloses and the composition of their monomeric sugar followed the alditol acetate method described^54^. The alcohol extractive-free samples firstly were treated with amylase and pullulanase (Sigma) in a 50 mM sodium acetate buffer (pH 4.8) overnight to remove starch. The de-starched material then was hydrolyzed with 2 M TFA at 121 °C for 60 min. The released sugars were reduced to their corresponding alditols by adding 0.5 M freshly prepared sodium borohydride solution (in dimethyl sulfoxide) at 40 °C for 90 min. After neutralizing with acetic acid, the alditols were incubated with acetic anhydride and 1-methylimidazole for acetylation. The resultant alditol acetates were finally dissolved in dichloromethane and separated on a gas chromatograph that was equipped with a 30 m × 0.25-mm (internal diameter) Agilent J &W HP-5MS capillary column, and the eluant introduced into a mass spectrometer (Agilent Technologies); the initial oven temperature was maintained at 38 °C for 30 s, increased to 170 °C at 50 °C min^−1^, and then increased to 230 °C at 2 °C min^−1^ and held there for 5 min. The individual sugars were identified by comparison with authentic standard compounds; their quantitation was based on the standard curves of each derivatized individual sugar made from the same GC–MS run.

### FT-IR spectroscopic imaging

Infrared imaging of the sections of poplar stems followed the method described by Gou *et al.*^55^ Briefly, a piece of aspen stem from ∼1 month-old plantlet within the first five internodes was cut and fresh-frozen over liquid nitrogen. Then, the specimen was embedded in Tissue-Tek freezing medium and sectioned with a cryomicrotome (Leica Microsystems) at −20 °C. Sections (10 μm thick) were placed on a BaF2 microscope slide and dried at room temperature. IR images were collected using a Perkin Elmer Spectrum Spotlight FT-IR Imaging System (Waltham, MA, USA) with a 6.25-μm pixel resolution. For each tissue section, a light micrograph was obtained, and the regions to be imaged with the IR microscope were defined. Infrared images were collected in the transmission mode by raster-scanning the sample through the IR beam, and collecting infrared absorbance spectra at each pixel. For each pixel, an entire infrared spectrum is obtained from 4,000- to 720-cm^−1^. Background spectra were gathered from a clean region of the BaF_2_ slide. The spectral resolution was 8 cm^−1^, and 16 scans were averaged for each spectrum.

The corresponding FT-IR image was created by visualizing the intensity of the spectra at 1,510 cm^−1^, which was assigned to the vibration of the phenolic ring from lignin^56^ and the spectra bands from 900- to 1,180-cm^−1^, representing the overlapped polysaccharides. Both sets of images were normalized to the C–H intensity to correct for any variation in sample thickness; empty pixels covering cells' lumens were eliminated by setting a threshold limit for the absorbance at lignin and polysaccharides.

### Determination of lignin molecular weight

The lignin fractions were prepared essentially as described^57^ with minor modifications. Briefly, the prepared poplar cell walls were further intensively milled using a Retsch ball mill PM100 for 5 × 10 min with 5 min pause in between. The ball-milled materials were extracted twice with 96:4 dioxane:water. The extracts were pooled and freeze-dried, resulting in the milled wood lignin (MWL). The insoluble materials were then treated at 30 °C with a crude cellulase mixture (Onozuka R-10, PhytoTechnology Laboratories). The materials were extracted twice more with 96:4 dioxane:water. The extracts were pooled and freeze-dried, giving rise to so-called cellulolytic enzyme lignins (CEL). The remaining insoluble materials were referred to as residue lignin (RL). The different lignin materials were then dissolved in *N*-methylimidazole (NMI) and dimethyl sulfoxide in a ratio of 1:2 and acetylated by adding acetic anhydride (0.6 × NMI)^58^. Analyses of the molecular weight for different lignin fractions were performed by HPLC with a PHENOGEL Linear (2) column (00H-3259-K0) using tetrahydrofuran as the mobile phase. The flow rate was set at 0.5 ml min^−1^.

### Fermentation of aspen wood

Basal stems (∼30 cm) from 6-month-old newly propagated *MOMT4* transgenic and control plantlets, or from ∼1-year-old primary transgenic trees after three coppice events were harvested, air dried and milled to fine powder (passing through a 60-μm mesh sieve). The materials were used directly for fermentation or were pretreated as follows: the wood powders were mixed with 1% Ca(OH)_2_ at the ratio of 10% (w/v) biomass, and treated at 121 °C for 6 h. The treated powders were collected by centrifuging, and then washed three times using distilled water in 2 ml ddH_2_O/g wood biomass to remove the soluble materials. The pretreated wood powders then were completely dried at 45 °C. Simultaneous saccharification and fermentation was undertaken following an established protocol^6^ with slight modification. Briefly, the fermentation was carried out in capped aseptic bottles containing 8% (w/v) woody biomass (either pretreated or untreated), 1% (w/v) yeast extract, and 2% (w/v) bactopeptone in 50 mM sodium citrate buffer (pH 4.8). After autoclaving them, we added accellerase 1500 (DuPont, WI, USA) that contains multiple enzyme activities, including endoglucanase (2,200–2,800 CMC U g^−1^), and β-glucosidase (450–775 pNPG U g^−1^), at 0.3 ml g^−1^ biomass, and yeast (*Saccharomyces cerevisiae* D5A, ATCC 200062) at a final OD_600_ of 0.5 in the total fermentation reaction volume of 12.5 ml. The broth was incubated at 37 °C in a shaker at 175 r.p.m. to prevent sedimentation of the substrate, and the yeast. At the indicated incubation time, we recorded the weight of the aseptic bottle containing the fermentation broth and then the bottle was vented using a hyperdermic needle. After allowing for adequate equilibration of the pressure and temperature of the bottle (about 30 min), the needle was removed from the bottle and the bottle was weighed again. The difference of the weight before and after ventilation represents the weight loss (that is, gas release) of fermentation. Then, an aliquot of broth (∼20 μl) was taken from the bottles; after centrifuging, the supernatant was collected and stored for later determining its ethanol content. Ethanol was detected and quantified with Agilent GC7690A equipped with a capillary column (Alltech EC-WAX, 30 m × 0.32 mm × 0.25 μm, Econo-Cap, GRACE), and a flame-ionization detector. The analysis was carried out under the following conditions: Oven temperature, 45 °C for 6 min, then programmed to 180 °C at a rate of 35 °C min^−1^, and held there for 2 min; the temperature of both the injector and detector was 225 °C; the nitrogen (carrier gas) total flow rate was 14 ml min^−1^; the hydrogen flow rate was 30 ml min^−1^; and the air flow rate was 400 ml min^−1^., and the flow rate of the column gas was 1 ml min^−1^; Samples were diluted 10-fold; the injection volume was 1 μl, with a split ratio of 25:1. n-Propanol was used as an internal standard for calibration. We used the standard curve of ethanol made in the same GC-FID run for quantifying the yield of fermentation ethanol.

### Wood density

Approximately 8-month-old control plants and MOMT4 primary transgenic plants were harvested. After removing their bark, the primary stems then were dried at 45 °C. The basal part of the stem of each line was then cut into three sections (each ∼5 cm), the dry weight (Ws) of each section was measured first; the volume of each section was then measured in a graduated cylinder filled with fine glass beads (soda lime with diameter at 0.5 mm, Biospect Prodcuts). The volume of glass beads before and after inserting stem section was recorded and termed *V*0 and *V*1, respectively. The volume of each stem was calculated with *V*s*=V*1−*V*0. The density of the wood was calculated as *D*s*=W*s/(*V*1−*V*0). The data from six control lines and eleven transgenic lines were averaged, respectively.

### Scanning electron microscopy

The 16th nodes from the top of 2-month-old aspen shoots of *MOMT4* transgenic- and control lines were fixed in 2.5% glutaraldehyde and 1% formaldehyde in 0.2 M sodium phosphate buffer (pH 7.0). Thereafter, the samples were washed with the buffer three times and the lower 1 cm-stem segments of both the transgenic- and the control lines were prepared and cut at 50 μm with a Leica VT 1000 S vibratome (Leica Microsystems, Heidelberg, Germany). Five sections each from the transgenic- and control-lines were prepared and imaged with optical microscopes. The remaining stem segments were then dehydrated in a series of graded ethanol solution and dried with a Polaron critical-point drying apparatus (Polaron Instruments Inc., Doylestown, PA, USA). The samples were mounted on aluminium stubs, sputter-coated with gold, and the vibratome cutting faces were imaged at 25 kV and in high vacuum mode with a Quanta 200 environmental scanning electron microscope (FEI Company, Hillsboro, OR, USA).

### Enzyme activity inhibition assay

To prepare 4-*O*-methylated monolignols, the purified MOMT4 enzyme was incubated in a reaction mixture containing 50 mM Tris-HCl (pH 7.5), 2 mM coniferyl alcohol or sinapyl alcohol, 10 mM SAM and 1 mM DTT. The reaction proceeded at 30 °C for 2 h to produce 4-*O*-methylated coniferyl alcohol (4OMeCA) or 4-*O*-methylated sinapyl alcohol (4OMeSA). After extraction with water-saturated ethyl acetate, the organic phase was dried under stream of nitrogen gas. The purity of products was examined by HPLC, and the authenticity was confirmed by LC–MS.

For poplar gene cloning and enzyme preparation, the total RNAs were extracted using a CTAB-based method^59^ from wild-type hybrid aspen. The first strand cDNA was synthesized using M-MuLV Reverse Transcriptase (New England Biolabs) under the manufacture recommended conditions. Cinnamyl alcohol dehydrogenase genes *PtrCAD* and *PtrSAD,* and P450 membrane-bound enzyme gene *PtrF5H* were amplified by RT-PCR, respectively, using primers listed in Supplementary Table 5. The PCR products were then subcloned into pCR8-GW/TOPO vector with the standard procedure (Invitrogen) to obtain the corresponding gateway cloning entry vectors. The constructs were verified by sequencing. *PtrCAD* and *PtrSAD* were then introduced by the LR reaction into a gateway-compatible vector, pETG-41K (http://www.helmholtz-muenchen.de), and transformed into BL21-CodonPlus (DE3)-RIPL (Agilent). The recombinant proteins were purified by Ni-NTA affinity chromatography. The aspen stem's crude proteins (PtrSTEX) were extracted using the procedure described^60^ with minor modifications. Briefly, the developing stems of 3-month-old wild-type aspens were harvested and ground into a fine powder under liquid nitrogen. The powder was extracted with a 20 mM Tris–HCl (pH 7.5) buffer containing 10 mM ascorbate, 1.5% PVPP and 1 mM PMSF. After centrifuging at 10,000*g* for 20 min at 4 °C, the supernatant was filtered through a 0.45 μm membrane and stored at −70 °C for further use. *PtrF5H* was cloned into pYES-DEST52 and transformed into the yeast strain WAT11 for expression. Incubation of yeasts and induction of protein expression were performed by using high-density procedure^61^. After induction with 20% galactose for 18 h, yeast cells were harvested and disrupted with Zymolasa 100T (Seikagaku, Tokyo). After centrifuging at 12,000*g* for 10 min, the resulting supernatant were then ultra-centrifuged at 140,000*g* for 90 min. The microsomal pellet was resuspended in assay buffer (50 mM sodium Pipes (pH 7.0) containing 20% glycerol and 4 mM disodium EDTA) and stored at −80 °C. *PtrCOMT* was cloned from *Populus trichocarpa* and its recombinant enzyme was expressed and produced in *Escherichia coli* BL21(DE3) strain^62^.

The cinnamyl alcohol dehydrogenase activity was measured essentially as described^34^. Briefly, a 50 μl reaction contained 50 mM Tris–HCl (pH7.5), 1 mM β-mercaptoethanol, 0.5 mM NADPH, and 0.5 mM hydroxycinnamaldehyde. PtrCAD (1.5 μg), 5 μg PtrSAD, or 2 μg PtrSTEX was used in the reaction with sinapaldehyde as the substrate; 3 μg PtrCAD, 15 μg PtrSAD, or 10 μg PtrSTEX was used in the reaction with coniferaldehyde as the substrate. PtrF5H activity was measured according to Humphreys *et al.*^63^ using 1 mM coniferyl alcohol as a substrate in a 50 μl reaction containing 39.25 μl (29.7 mg protein per ml) microsome. For assaying COMT activity, the reaction mixture containing 100 mM Tris-Cl (pH 7.5), 1 mM DTT, 500 μM SAM, and 500 μM caffeic acid and 1.5 μg purified PtrCOMT or 20 μg PtrSTEX was incubated at 30 °C for 5 min. All the enzyme assays were conducted either in the presence or absence of 40 μM 4-*O*-metylated -coniferyl alcohol or -sinapyl alcohol to examine their potential inhibition on the activity. The enzymatic products were profiled using HPLC following the method described above.

### *In vitro* polymerization assay

500 μM coniferyl alcohol or sinapyl alcohol was incubated with 40 ng horseradish peroxidase (Sigma), and 1.14 mM H_2_O_2_ in 25 mM Tris-Cl buffer (pH 7.5) in the presence or absence of 40 μM 4OMeCA or 4OMeSA. The reaction was preceded for 10 min at 30 °C. The products were extracted by water-saturated ethyl acetate, dried under the stream of N_2_ gas, then re-dissolved in 50 μl of methanol and profiled by HPLC. A crude extract of aspen stem, PtrSTEX, was used for assaying peroxidase activity instead of HRP.

### qRT-PCR analyses of monolignol biosynthetic genes

The 4th–13rd internodes of 2-month-old developing stems were harvested from two independent control lines and two *MOMT4* transgenic lines (*MOMT4-0* and *MOMT4-2*). For each line, two individual plants were respectively sampled. The total RNAs were extracted using a CTAB-based method^59^. First strand cDNA was synthesized using M-MuLV Reverse Transcriptase (New England Biolabs) under the manufacture recommended conditions. The selection of monolignol biosynthetic genes and primer designs were performed as described^35^ except *PtrPT1* (ref. 64) was used as the reference gene. For each sample, qRT-PCRs were carried out in triplicates for all the genes examined with primers listed in Supplementary Table 5. SsoAdvanced Universal SYBR Green Supermix (Biorad) was used for the reaction. The cycle threshold (Ct) value was calculated by the CFXManager Software v3.0 (Biorad). Gene expression was analysed using the comparative Ct method against the reference gene *PtrPT1*.

### RNA sequencing

The 1st to 15th internodes from the top of 2-month-old hybrid aspen stems of MOMT4-0 transgenic and control lines were harvested and, after removing their barks, were immediately frozen in liquid nitrogen. About 100 mg stem samples pooled from 2 to 3 individual transgenic and control plants, respectively, were grounded to fine powder under liquid nitrogen. Total RNAs were extracted using Qiagen RNeasy Plant Mini Kit (#Cat. 74903) following the manufacturer recommended procedures. The assessment of purity and concentration of each RNA sample, and the strand-specific RNA-Seq library construction were conducted by using the Polar Genomics (Ithaca, NY, USA) service. Sequencing was done using the Illumina HiSeq2500 platform via 100-bp single-end reads of multiplexed RNA samples. The resulting RNA-Seq reads were aligned to the *P. trichocarpa* genome downloaded from the PlantGDB (http://www.plantgdb.org/XGDB/phplib/download.php?GDB=Pt) using TopHat^65^ allowing one mismatch. Following these alignments, raw counts for each poplar gene were derived and normalized to reads per kilobase of exon model per million mapped reads (RPKM). The differentially expressed genes were identified with the integrated Cuffdiff programme^66^,^67^ based on False Discovery Rate adjusted *p* value (that is, *q* value) at the cutoff of 0.05.

### Statistical analysis

Statistical analysis was performed by Student's *t*-tests (two-tail distribution and two-samples with unequal variances). Statistically significant differences were defined as *P*<0.05. Values in graphs were presented as means with s.d. or s.e. Microsoft Excel 2011 (Microsoft Corporation) were used for data management, statistical analysis, and graph generation.

### Data availability

RNA-seq data described in this study has been deposited in the GEO Database under accession code GSE81123. The authors declare that all other data supporting the findings of this study are available within the article and its Supplementary Information files or are available from the corresponding author upon request.

## Additional information

**How to cite this article**: Cai, Y. *et al.* Enhancing digestibility and ethanol yield of Populus wood *via* expression of an engineered monolignol 4-O-methyltransferase. *Nat. Commun.* 7:11989 doi: 10.1038/ncomms11989 (2016).

## Supplementary Material

Supplementary Information Supplementary Figures 1 - 7 and Supplementary Tables 1 - 5

## Figures and Tables

**Figure 1:**
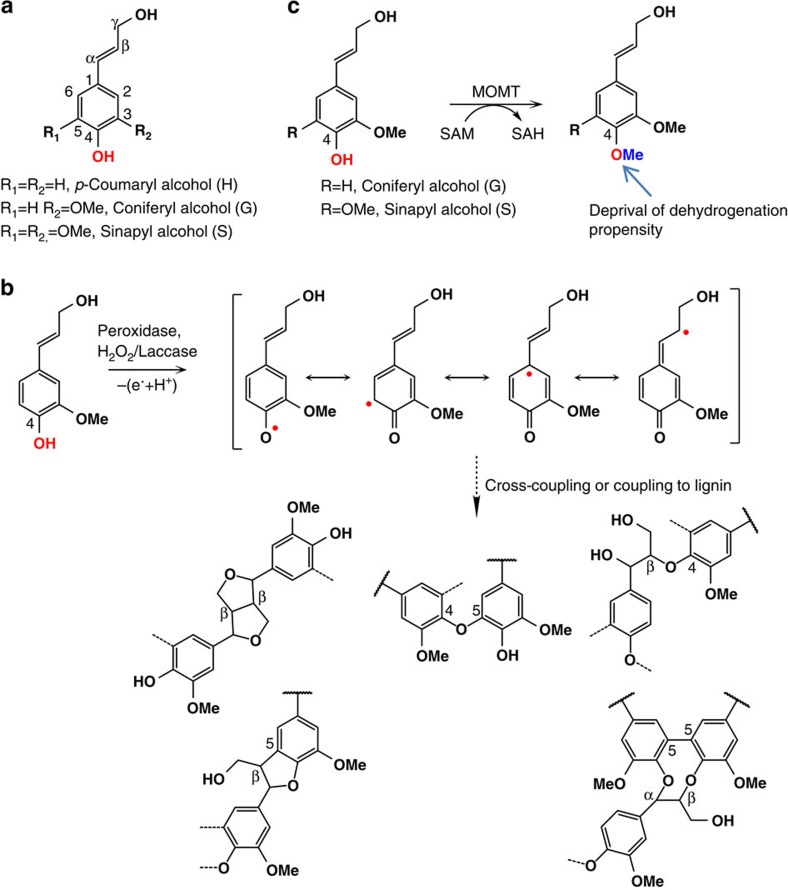
Illustration of MOMT4-medidated depression on lignin polymerization. (**a**) The conventional monolignol structures. (**b**) Scheme for monolignol oxidative dehydrogenation and polymerization processes. (**c**) The reaction catalysed by the engineered monolignol 4-*O*-methyltransferase, which deprives the propensity of the modified lignin precursors for further polymerization.
