# Supplementary material for: Enhancing digestibility and ethanol yield of Populus wood via expression of an engineered monolignol 4-O-methyltransferase
Source: Nat Commun. 2016 Jun 28;7:11989. doi: 10.1038/ncomms11989 (PMC4931242; doi:10.1038/ncomms11989)
Supplement: Supplementary Information — Supplementary Figures 1 - 7 and Supplementary Tables 1 - 5 [file ncomms11989-s1.pdf]

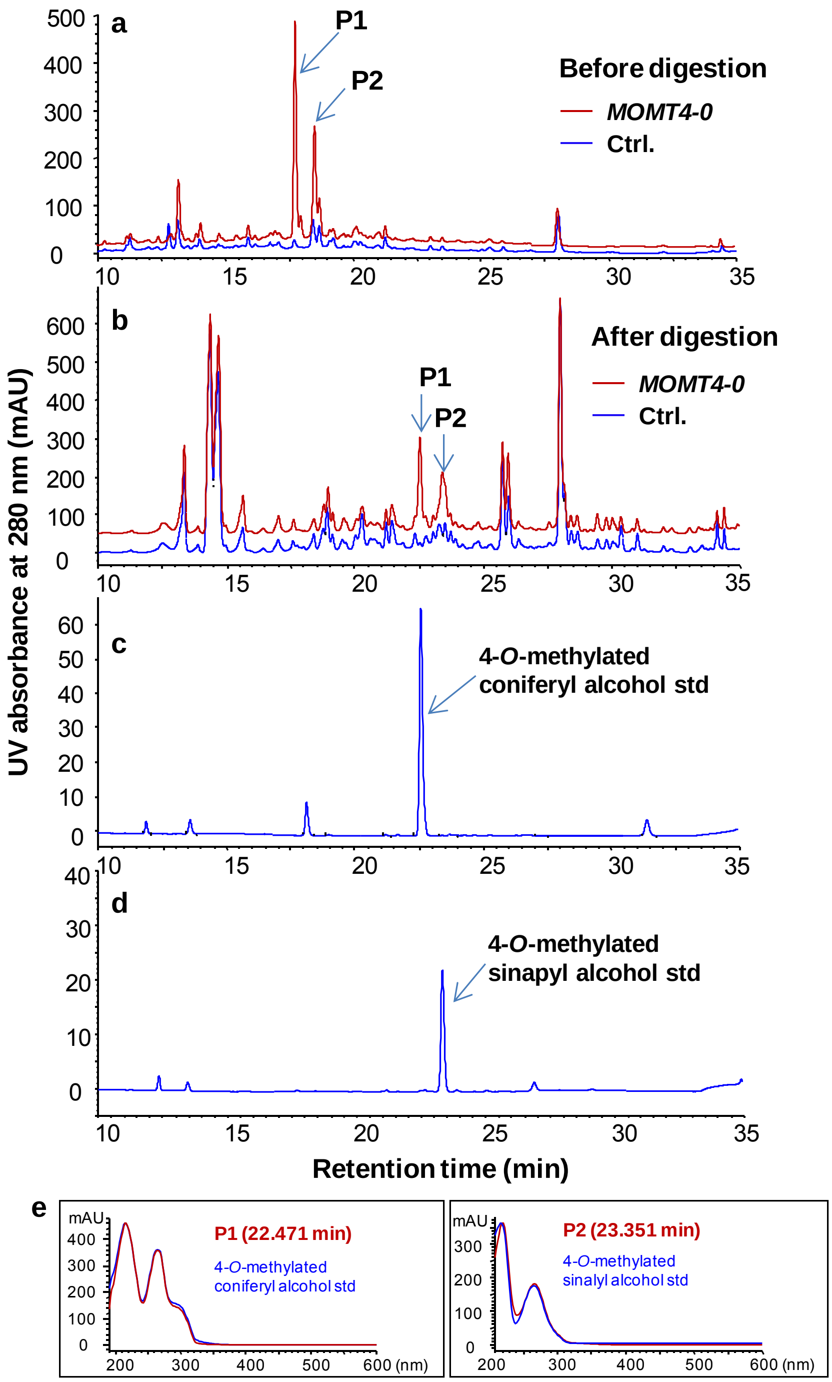

Supplementary Figure 1 **(Contl. on page 2)**

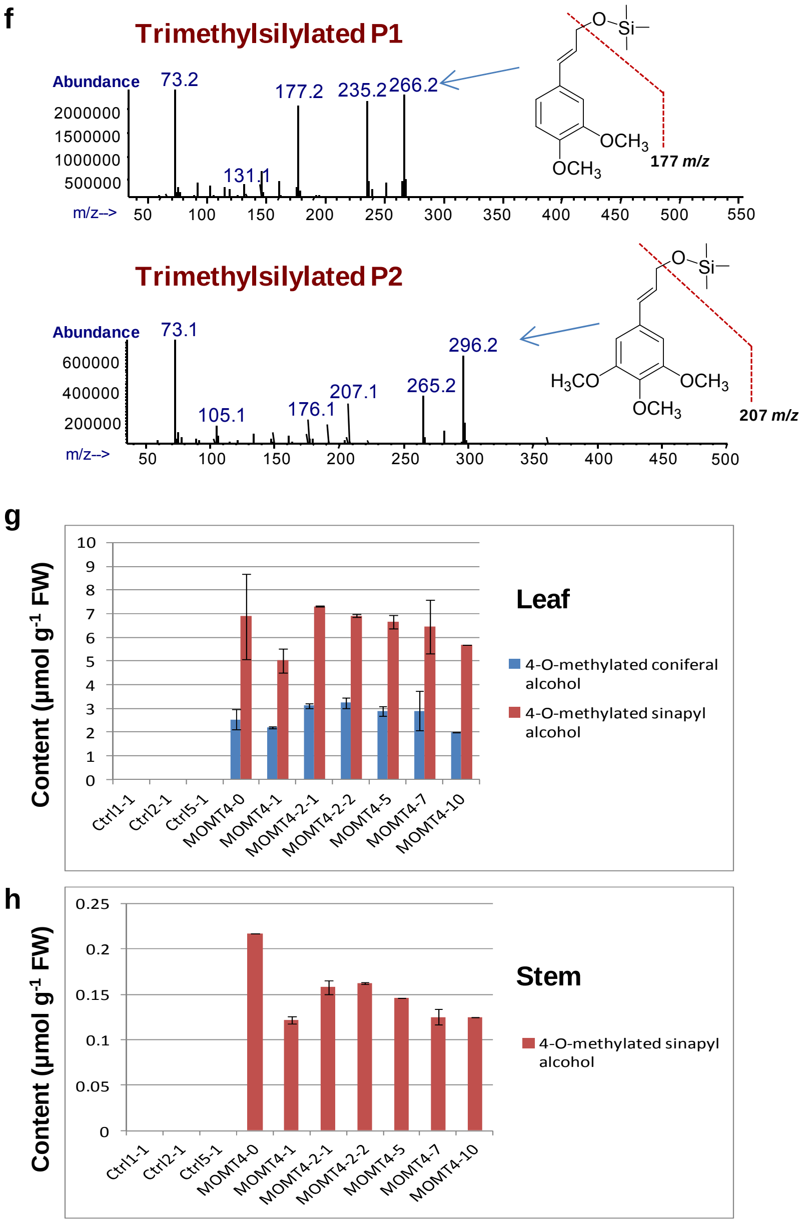

Supplementary Figure 1. Characterization of methanol extractable phenolics from the *MOMT4* transgenic and control aspens. (**a**) HPLC profiling of methanol-extractable phenolics from the leaves of *MOMT4-0* transgenic and control aspens before β-glucosidase digestion. (**b**) HPLC profiling of methanol-extractable phenolics in (**a**) after β-glucosidase digestion. Two unique compounds were detected in the *MOMT4-0* with P1 identical to the prepared authentic standard of 4-*O*-methylated coniferyl alcohol (**c**) and P2 identical to 4-*O*-methylated sinapyl alcohol standard (**d**). (**e**) UV spectra of P1 and P2 in (**b**), compared to those of the prepared standards. (**f**) Mass spectra of two detected unique metabolites in the leaf extracts of *MOMT4-0* (after β-glucosidase digestion). (**g**) Accumulation level of the 4-*O*-methylated monolignols in the 2-month-old aspen leaves. (**h**) Accumulation level of the 4-*O*-methylated monolignols in the 2-month-old stems; only 4-*O*-methylated sinapyl alcohol was detected in the transgenic stem extract. Data are mean ± s.d. from two experimental repeats. FW: Fresh weight.

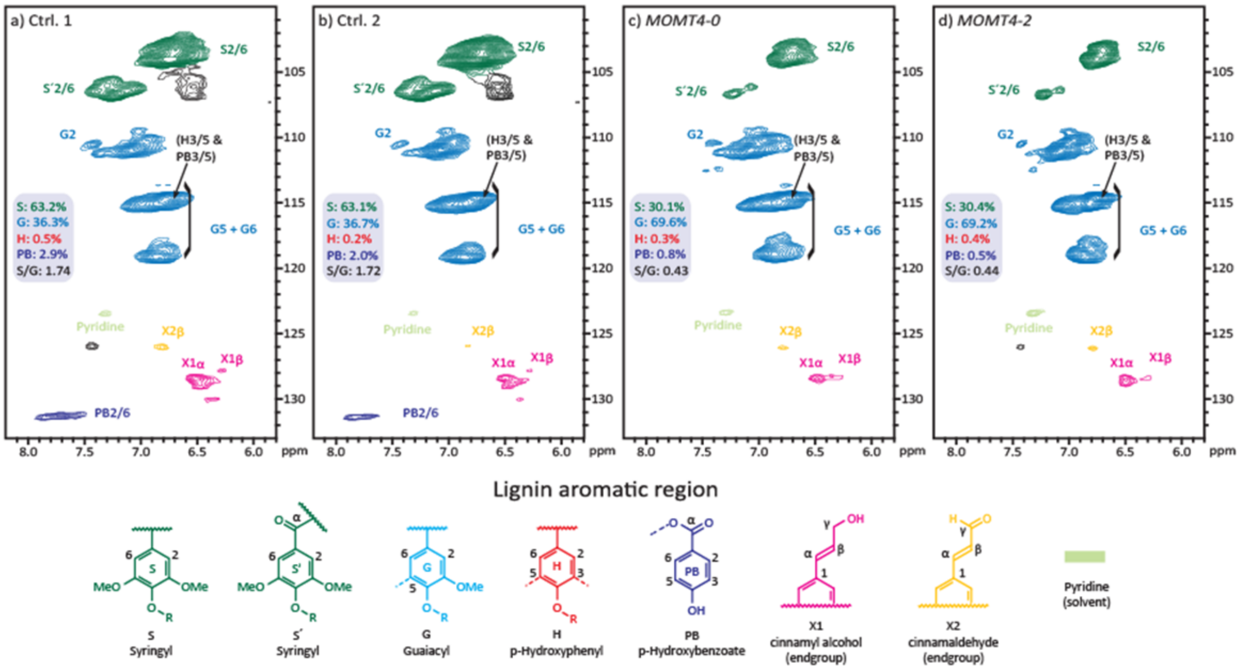

Supplementary Figure 2. Partial short range 2D HSQC NMR spectra of aromatic region. Equal amounts of solubilized total cell walls from two controls (**a** and **b**) and two *MOMT4* transgenic plants (*MOMT4-0 and MOMT4-2*) (**c** and **d**) were examined**.** The main structural units are colored to coincide with their structures at the bottom.

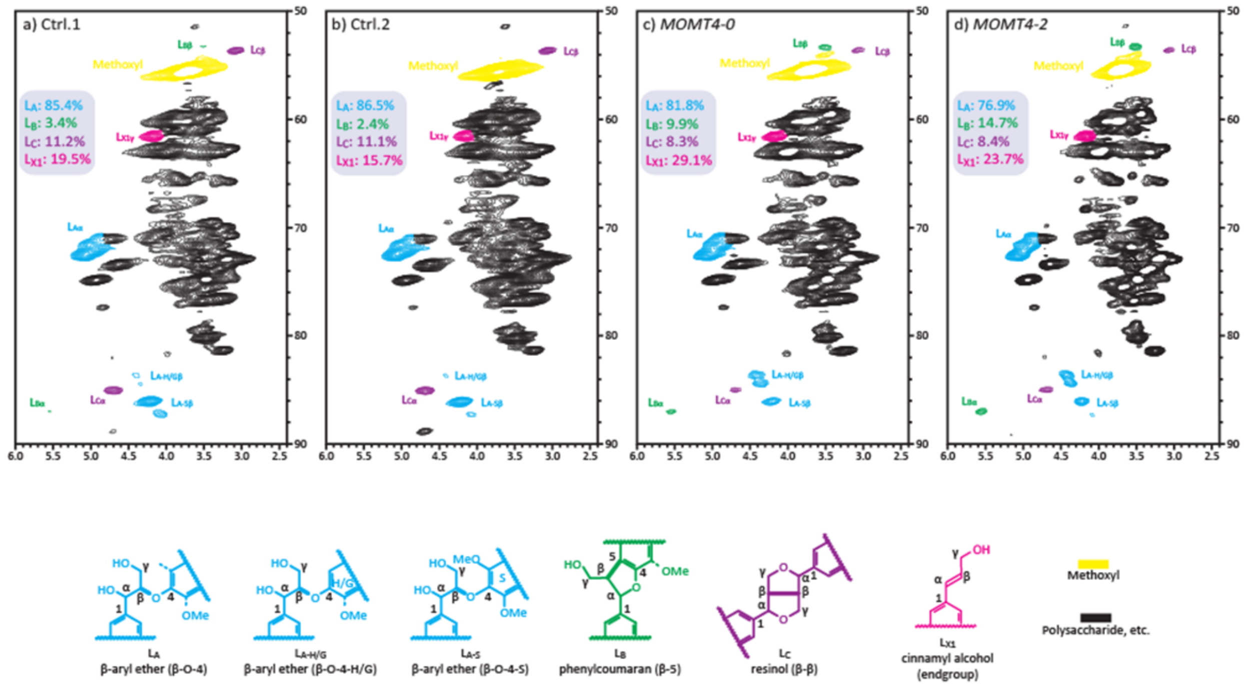

Supplementary Figure 3. Partial short range 2D HSQC NMR spectra of lignin aliphatic and polysaccharide region. Equal amounts of solubilized cell walls of two control (**a** and **b**) and two *MOMT4* transgenic aspens (**c** and **d**) were examined**.** The main units, characterized by their inter-unit linkages, are colored to coincide with their structures at the bottom.

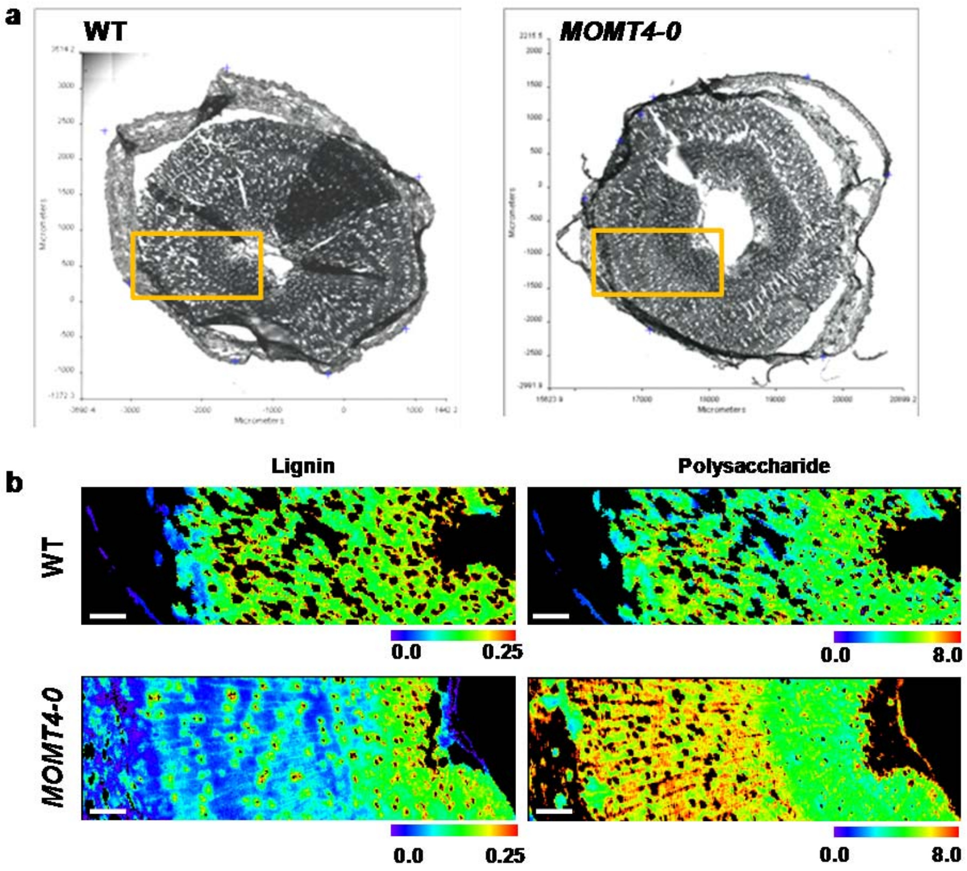

Supplementary Figure 4. Infrared imaging of a cross section of aspen stem and the crystalline cellulose content in *MOMT4* transgenic cell walls. (**a**) Light micrograph of a transverse section of hybrid aspen young stem. The frame illustrates the region imaged with the IR microscope. (**b**) Infrared images of the lignin (1510 cm^{-1}) and polysaccharides (900-1180 cm^{-1}) in aspen stem cross-section. The distribution of each component was generated by integrating the peaks centered at their characteristic IR absorptions. The content is shown as heat-map. Scale bar= 25 µm.

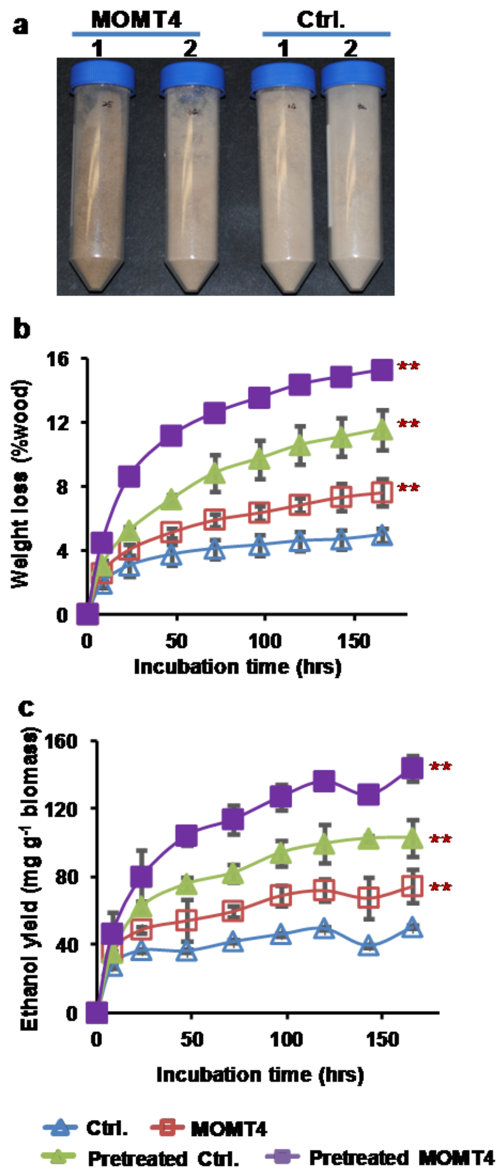

Supplementary Figure 5. Simultaneous saccharification and fermentation of about one year old *MOMT4-0* transgenic woods to ethanol. Wood samples were pretreated with 1% (w/v) $Ca(OH)_2$ and incubated at 121 °C for 6 h. The Wood samples were collected from ~1 year old basal stems of the primary transgenic lines that had been coppiced three times and were maintained in a greenhouse. (**a**) Pretreated wood samples. #1 and #2 represent two replicates. (**b**) Fermentative gas release (representing biomass weight loss in broth) during simultaneous saccharification and fermentation (SSF) of the pretreated and untreated control and *MOMT4-0* transgenic woods. (**c**) Ethanol yield from the pretreated and untreated control and *MOMT4-0* transgenic woods during SSF. Data represent mean ± s.d. of three experimental repeats. ** $P<0.01$ (Student's *t*-test) indicating significant difference compared to the control.

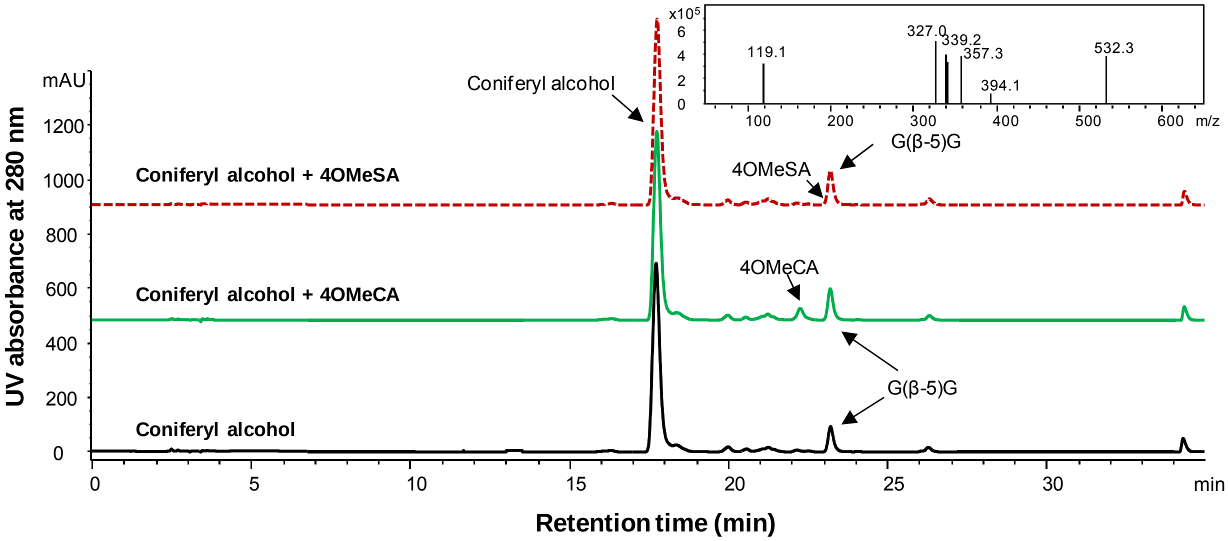

Supplementary Figure 6. LCMS profiles of the *in vitro* oxidative polymerization reactions of coniferyl alcohol mixed with 4-*O*-methyalted monolignols.
4-*O*-methyalted coniferyl alcohol (4OMeCA, green line) or 4-*O*-methylated sinapyl alcohol (4OMeSA, red line) were used, and reactions were catalyzed by peroxidase with H_2O_2. Note that no inhibition of 4OMeCA or 4OMeSA on the oxidative polymerization of the phenolic monomers was observed. Inset shows the MS spectrum of G(β–5)G dimer.

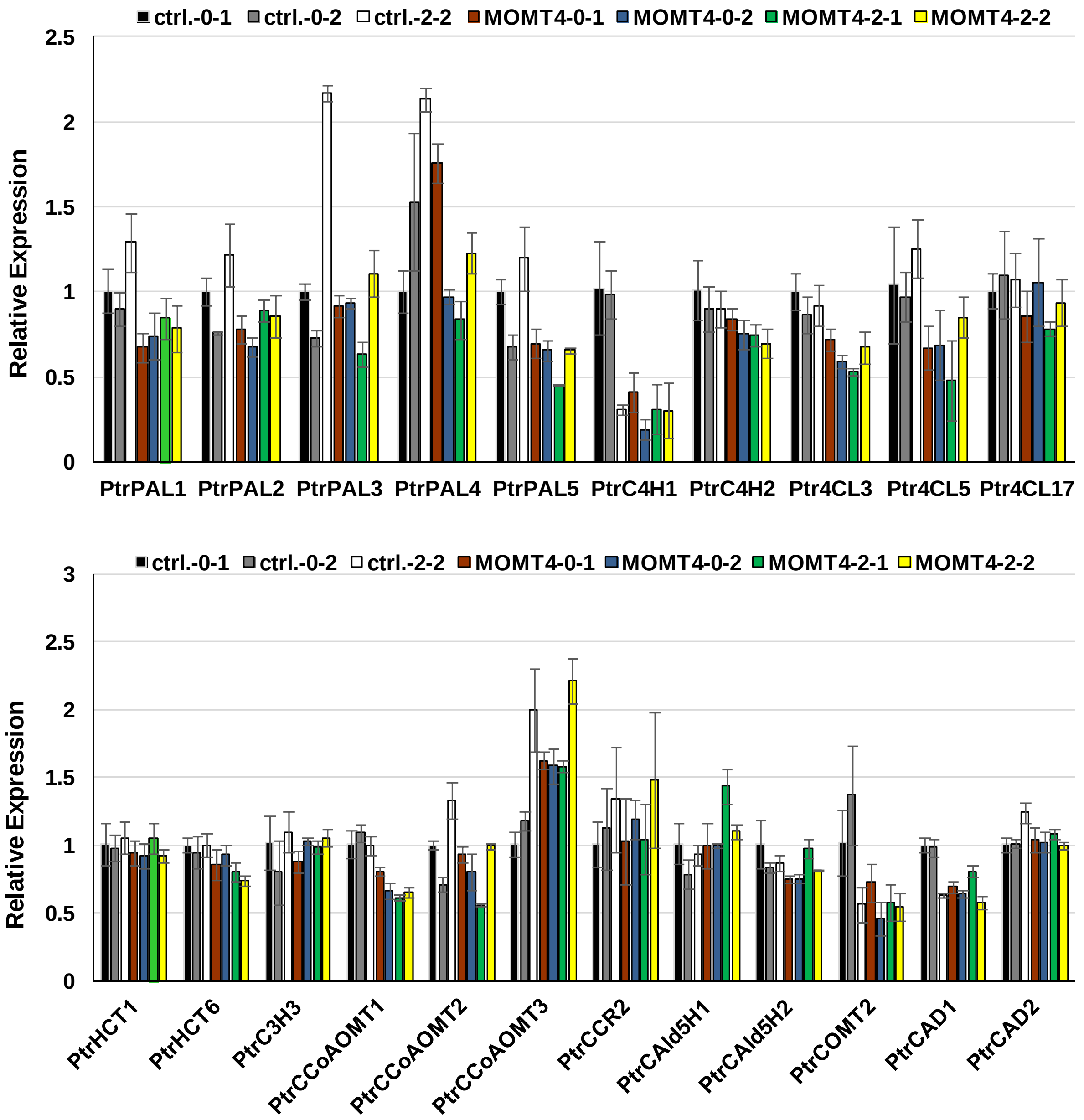

Supplementary Figure 7. Relative expression level of xylem specific monolignol biosynthetic genes in *MOMT4* transgenic and control lines.

Data were obtained by quantitative RT-PCR and represent mean ± s.d. of three replicates for each gene in each of control and *MOMT4* transgenic lines (each line with a biological duplicate). Data from one control line was set as 1.

Supplementary Table 1. Monomeric sugar composition of hemicelluloses extracted from *MOMT4* transgenic wood.

Genotype	Rhamnose (%)	Arabinose (%)	Xylose (%)	Mannose (%)	Glucose (%)	Galactose (%)	Total (%)
**Control**	0.54 ±0.01	0.29±0.01	9.38±0.62	10.60±0.93	1.26±0.06	0.53±0.01	22.60±1.64
**MOMT4-0**	0.58 ±0.04	0.31±0.02	9.12±0.75	8.70±1.60	1.43±0.12	0.59±0.03	20.73±2.39
**MOMT4-2**	0.55±0.01	0.29±0.01	11.32±1.41	8.71±1.94	1.35±0.22	0.53±0.02	22.74±3.07
**MOMT4-7**	0.57±0.02	0.31±0.01	10.13±0.5	9.98±2.29	1.60±0.28	0.55±0.03	23.15±2.83
**MOMT4-10**	0.55±0.01	0.28±0.01	8.3±0.2	13.88±0.56*	2.04±0.08**	0.53±0.01	25.55±0.79

The values are expressed as % of cell wall residues and represent mean ± s.e. from three biological replicates (each with three technical repeats) for control set and three technical repeats for each transgenic line. Statistic analysis was performed with Student's *t*-test (* $P<0.05$, ** $P<0.01$)

Supplementary Table 2. The stem height, basal stem diameter, and biomass yield of 3 or 6 month old aspen plants.

	3 months		6 months		
	Stem height (cm)	Diameter (cm)	Stem height (cm)	Diameter (cm)	Biomass (g)
**Control**	90.6±12.8	0.85±0.07	254.7±42.5	1.78±0.22	120.4±22.5
**MOMT4-0**	94.8±8.9	0.92±0.03	247.3±23.2	1.84±0.19	112.5±24.9
**MOMT4-2**	77.9±7.3	0.81±0.04	209.7±16.8	1.81±0.03	105.5±8.6
**MOMT4-7**	91.4±11.6	0.9±0.01	233.0±19.9	1.72±0.08	107.7±11.2

Data represent mean ± s.d. of three biological replicates for each of control and transgenic line. Student’s *t*-test reveals no statistic difference between the control and individual *MOMT4* transgenic line.

Supplementary Table 3. **Effects of 4-*O*-methylated coniferyl alcohol and 4-*O*-methylated sinapyl alcohol on the activities of enzymes involved in sinapyl alcohol biosynthesis.**

Substrate	Enzyme activity (nmol·min^{-1}·mg^{-1})		
	Ctrl.	4OMeCA	4OMeSA
**coniferaldehyde**			
PtrCAD	96.97±2.37	87.82±1.32**	88.60±1.42**
PtrSAD	23.29±0.24	23.34±0.83	22.99±0.93
PtrSTEX	83.95±0.21	82.90±0.17*	85.82±2.64
**sinpaldehyde**			
PtrCAD	310.55±6.40	265.72±1.95**	299.12±10.65
PtrSAD	203.23±8.65	184.50±4.73*	175.83±2.32**
PtrSTEX	182.5±0.59	163.93±1.02**	182.57±6.90
**caffeic acid**			
PtrCOMT	424.42±6.12	434.36±3.57	433.78±3.98
PtrSTEX	4.55±0.02	4.66±0.03**	4.58±0.01
**coniferyl alcohol**			
PtrCAld5H/WAT11	2.28±0.18[a]	2.41±0.09[a]	2.45±0.23[a]

Data represent mean ± s.d. of triplicates. * P<0.05; ** P<0.01 (Student's *t*-test); 4OMeCA: 4-*O*-methylated coniferyl alcohol; 4OMeSA: 4-*O*-methylated sinapyl alcohol

[a.]pmol·min^{-1}·mg^{-1}

Supplementary Table 4. The up- and down-regulated genes in *MOMT4-0* transgenic line detected with RNA-sequence analysis. The differentially expressed genes with Log$^2 \geq 1$ and $\leq$-1 and with False Discovery Rate adjusted P<0.05 between *MOMT4-0* transgenic and control plants were selected.

Test_ID	Orthologue gene_ID	Arabidopsis annotation	WT	MOMT4	Log^2(MOMT4/WT)
MyGene	MOMT4	Over-expressed gene	1221.8	4.78528	7.996189
Potri.001G024900	AT2G38750.1	Annexin 4	24.6869	1.62984	3.920943
Potri.019G031200	AT5G18280.2	Apyrase 2	20.8676	1.96577	3.408098
Potri.013G080300	AT4G24340.1	Phosphorylase superfamily protein	33.9166	4.39997	2.946426
Potri.008G131200	AT1G14930.1	Polyketide cyclase/dehydrase and lipid transport superfamily protein	169.477	22.9856	2.882287
Potri.014G126900	AT4G02290.1	Glycosyl hydrolase 9B13	12.5103	2.47025	2.340387
Potri.004G106600	AT3G25180.1	Cytochrome P450,CYP82G1	37.2716	9.11907	2.031118
Potri.013G080400	AT4G24350.1	Phosphorylase superfamily protein	83.5143	20.6367	2.016811
Potri.004G123200	AT1G69530.1	Expansin A1	33.3327	8.63563	1.948565
Potri.018G031100	AT5G11420.1	Protein of unknown function, DUF642	103.209	28.1813	1.872759
Potri.014G126000	AT5G64260.1	EXORDIUM like 2	62.808	17.6666	1.829924
Potri.004G168600	AT4G38770.1	Proline-rich protein 4	28.2242	8.0906	1.802614
Potri.012G032700	AT1G74100.1	Sulfotransferase 16	26.7991	7.77299	1.785643
Potri.006G245600	AT4G13340.1	Leucine-rich repeat (LRR) family protein	51.6807	15.5388	1.733751
Potri.017G085300	AT3G29030.1	Expansin A5	18.4512	5.87431	1.651223
Potri.006G065500	AT2G10940.1	Bifunctional inhibitor/lipid-transfer protein	173.155	55.3255	1.646048
Potri.004G051900	AT5G45950.1	GDSL-like Lipase/Acylhydrolase superfamily protein	19.319	7.08953	1.446259
Potri.006G099900	AT3G51860.1	Cation exchanger 3	25.587	9.9398	1.364122
Potri.019G055200	AT1G08450.1	Calreticulin 3	78.6247	31.3292	1.327475
Potri.013G033200	AT1G24140.1	Matrixin family protein	37.1626	15.3381	1.276732
Potri.016G113100	AT2G38110.1	Glycerol-3-phosphate acyltransferase 6	22.2598	9.33544	1.253651
Potri.019G050200	AT4G24340.1	Phosphorylase superfamily protein	219.761	93.7438	1.22914
Potri.007G100100	AT5G09220.1	Amino acid permease 2	37.4223	88.542	-1.24246
Potri.005G172400	AT1G37130.1	Nitrate reductase 2	10.2372	24.5262	-1.2605
Potri.005G200400	AT5G09970.1	Cytochrome P450, CYP78A7	11.562	31.7595	-1.4578
Potri.005G014300	AT1G09240.1	Nicotianamine synthase 3	28.5745	78.9822	-1.4668
Potri.006G223900	AT5G12020.1	17 kDa class II heat shock protein	21.8466	79.3612	-1.86103
Potri.004G140800	AT2G15620.1	Nitrite reductase 1	15.3092	66.3609	-2.11593

Supplementary Table 5. Primers used in the study

Name	Sequence(5'-3')	Reference Gene ID
**For cloning bean PAL2 Promoter**		
PAL2_HindIII F	CCCAAGCTTAAAAGTCTAAGCCAA	
PAL2_KpnI R	GGGGTACCATGAAGGAATGA	
**For cloning poplar genes**		
PtrCADSLICF	ACAAAAAAGCAGGCTCCGAATTCATGGGTAGCCTTGAAACAG	POPTR_0009s09870g
PtrCADnsSLICR	ACAAGAAAGCTGGGTCGAATTCGGGAATAAGCTTGCTACC	
PtrSADSLICF	ACAAAAAAGCAGGCTCCGAATTCATGTCCAAGTCACCAGAAG	POPTR_0016s07910g
PtrSADnsSLICR	ACAAGAAAGCTGGGTCGAATTCGGGCTTCGTAGCTGCCAAAGT	
PtrF5HSLICF	ACAAAAAAGCAGGCTCCGAATTCATGGATTCTCTTGTCCAATC	POPTR_0007s13720g
PtrF5HnsSLICR	ACAAGAAAGCTGGGTCGAATTCGAGAGGGCATAGCACACG	
**For qRT-PCR analysis**		
PtrPAL1RTF	CCATCCAGGTCAAATTGAGGCTGCT	Potri.006G126800
PtrPAL1RTR	ACTTCTTAGCTGCCTTCATGTAAGCT	
PtrPAL2RTF	CCTAGAAGCCATCACCAAGTTGCTC	Potri.008G038200
PtrPAL2RTR	GTTTCTCCATTGGGTCCCACG	
PtrPAL3RTF	CATCCAGGTCAAATTGAGGCTGCA	Potri.016G091100
PtrPAL3RTR	ACTTCTTAGCTGCCTTCATGTAAGC	
PtrPAL4RTF	GAGATGCTGGAAGCTATCACCAAAT	Potri.010G224100
PtrPAL5RTF	GAGATGCTGGAAGCTATCACCAAGC	Potri.010G224100
PtrPAL4/5RTR	GGCTCTCCATTGGGTCCAACT	
PtrC4H1RTF	AGTGCGCCATAGACCATATCCTC	Potri.013G157900
PtrC4H1RTR	ATTGCAGCGACGTTGATGTTCTCA	
PtrC4H2RTF	GaAATGTGCAATTGATCATATTTTG	Potri.019G130700
PtrC4H2RTR	ATTGCAGCAACATTGATGTTCTCC	
Ptr4CL3RTF	ACTAGCCCATCCAGAGATATCCGA	Potri.001G036900
Ptr4CL3RTR	TCATCTTCGGTGGCCTGAGACTTT	
Ptr4CL5RTF	GTGATCATGCTCATCCTGCCAAGT	Potri.003G188500
Ptr4CL5RTR	TTGGCAGCAGTAGTAATGGCACCT	
PtrC3H3RTF	GTATGACCTTAGTGAAGACACAATCAT	Potri.006G033300
PtrC3H3RTR	CCCTTGGGTTCTTGATTAGCTC	
PtrHCT1RTF	ATCAGCATGTAAGGCACGCGG	Potri.003G183900
PtrHCT1RTR	TGCCAAAGTAACCAGGTGGAAGCGT	
PtrHCT6RTF	AGATCAACATGCAAAGCACGTGA	Potri.001G042900
PtrHCT6RTR	GCCAAAGTAACCAGGAGGGAGTTG	
PtrCCoAOMT1RTF	CAGTAATTCAGAAAGCTGGTGTTGC	Potri.009G099800
PtrCCoAOMT1RTR	GCATCCACAAAGATGAAATCAAAAC	
PtrCCoAOMT2/3RTF	CCTTCCAACGCCAGGAAAGAGAGTA	
PtrCCoAOMT2RTR	GTGGCCAACTTCTTGATGCCTTCCG	Potri.001G304800
PtrCCoAOMT3RTR	TGCACACAGCAACCATAGAGGACA	Potri.008G136600

PtrCCR2RTF	CGGTGATTCAGAAAGCTGGTCTGGA	Potri.003G181400
PtrCCR2RTR	GCATCCACAAAGATGAAGTCATAAG	
PtrCAld5H1RTF	AATCCAATATAGGCAAGCCTGTGAACG	Potri.005G117500
PtrCAld5H1RTR2	ATTTTTGGCCCCAAAAGCTGCTCTA	
PtrCAld5H2RTF	AAGCCAATATAGGCAAGCCTGTGAATC	Potri.007G016400
PtrCAld5H2RTR	ATTTTTAGCCCCGAAAGCTGCTCTG	
PtrCOMT2RTF	TCTTGAAGAATTGCTATGACGCCT	Potri.012G006400
PtrCOMT2RTR	GAATGCACTCAACAAGTATCACCTTG	
PtrCAD1RTF	GGCAAGCTGATCTTGATGGGTGTT	Potri.009G095800
PtrCAD1RTR	TCCCGGTGATTGACTTTCTCCCAA	
PtrCAD2RTF	AGTGACAGAAGTTGGGAGCAAGG	Potri.016G078300
PtrCAD2RTR	AGTGACATGCACCAACCAAGCATC	
PtrPT1RTF	GCGGAAAGAAAAACTGCAAG	POPTR_0014s03160
PtrPT1RTR	TGACAGCACAGCCCAATAAG	
Ptr4CL17RTF	CATGCCTGTGTCGCCAACCATTAT	POPTR_0012s09670g
Ptr4CL17RTR	GCAACTGAACCTCCGCAATGAACT	
PtrCOMT24RTF	ACTGTCGGAAAGCAATGCCTGAGA	POPTR_0019s13400g
PtrCOMT24RTR	TTGGCCATTTCCTTCTGGTTGCAG	
